# Single-base editing in *IGF2* improves meat production and intramuscular fat deposition in Liang Guang Small Spotted pigs

**DOI:** 10.1186/s40104-023-00930-4

**Published:** 2023-11-02

**Authors:** Tianqi Duo, Xiaohong Liu, Delin Mo, Yu Bian, Shufang Cai, Min Wang, Ruiqiang Li, Qi Zhu, Xian Tong, Ziyun Liang, Weilun Jiang, Shiyi Chen, Yaosheng Chen, Zuyong He

**Affiliations:** 1 State Key Laboratory of Biocontrol, School of Life Sciences, Sun Yat-Sen University, Guangzhou, 510275 Guangdong China; 2 Department of Pharmacology (the State-Province Key Laboratories of Biomedicine-Pharmaceutics of China, Key Laboratory of Cardiovascular Research, Ministry of Education), College of Pharmacy, Harbin Medical University, Harbin, 150081 China; 3 Hunan Provincial Key Laboratory for Genetic Improvement of Domestic Animal, College of Animal Science and Technology, Hunan Agricultural University, Changsha, 410128 China

**Keywords:** CBE3, IGF2, Intramuscular fat, Meat production, PI3K-AKT/AMPK, ZBED6

## Abstract

**Background:**

Chinese indigenous pigs are popular with consumers for their juiciness, flavour and meat quality, but they have lower meat production. Insulin-like growth factor 2 (IGF2) is a maternally imprinted growth factor that promotes skeletal muscle growth by regulating cell proliferation and differentiation. A single nucleotide polymorphism (SNP) within intron 3 of porcine *IGF2* disrupts a binding site for the repressor, zinc finger BED-type containing 6 (ZBED6), leading to up-regulation of *IGF2* and causing major effects on muscle growth, heart size, and backfat thickness. This favorable mutation is common in Western commercial pig populations, but absent in most Chinese indigenous pig breeds. To improve meat production of Chinese indigenous pigs, we used cytosine base editor 3 (CBE3) to introduce *IGF2*-intron3-C3071T mutation into porcine embryonic fibroblasts (PEFs) isolated from a male Liang Guang Small Spotted pig (LGSS), and single-cell clones harboring the desired mutation were selected for somatic cell nuclear transfer (SCNT) to generate the founder line of *IGF2*^*T/T*^ pigs.

**Results:**

We found the heterozygous progeny *IGF2*^*C/T*^ pigs exhibited enhanced expression of *IGF2*, increased lean meat by 18%–36%, enlarged loin muscle area by 3%–17%, improved intramuscular fat (IMF) content by 18%–39%, marbling score by 0.75–1, meat color score by 0.53–1.25, and reduced backfat thickness by 5%–16%. The enhanced accumulation of intramuscular fat in *IGF2*^*C/T*^ pigs was identified to be regulated by the PI3K-AKT/AMPK pathway, which activated SREBP1 to promote adipogenesis.

**Conclusions:**

We demonstrated the introduction of *IGF2*-intron3-C3071T in Chinese LGSS can improve both meat production and quality, and first identified the regulation of IMF deposition by *IGF2* through SREBP1 via the PI3K-AKT/AMPK signaling pathways. Our study provides a further understanding of the biological functions of IGF2 and an example for improving porcine economic traits through precise base editing.

**Supplementary Information:**

The online version contains supplementary material available at 10.1186/s40104-023-00930-4.

## Background

Over the past few decades, high-intensity selection for lean growth rate in pig breeds through conventional breeding strategy has resulted in reduction in meat quality, especially in reduced intramuscular fat (IMF) deposition [1]. China possess a variety of indigenous pig breeds with excellent meat quality, especially in IMF content [2]. However, most of them perform poor in lean growth rate, which substantially affects their commercial values. Therefore, precise modification of genes or loci with large genetic effects on lean growth rate while maintaining their excellent meat quality is expected to greatly improve the commercial value of Chinese indigenous pig breeds.

Insulin-like growth factor 2 (IGF2), the first identified maternally imprinted growth factor across different species, is exclusively expressed from the paternal copy [3], under the control by multiple promoters. IGF2 is strongly expressed in the embryonic stage, and less expressed in adult tissues. It plays an important role in muscle growth by regulating myoblast proliferation, apoptosis and differentiation [4, 5]. Zinc finger BED-type containing 6 (ZBED6), represses the normal transcription of *IGF2* by binding to the conserved motif 5'-GCTCG-3' located within a CpG island in intron 3 of *IGF2* gene [6, 7]. In Western pig breeds, the existence of natural *IGF2*-intron3-G3072A substitution can abolish the binding of ZBED6 on motif, increase *IGF2* mRNA levels more than threefold, leading to increased lean meat mass in ham by 5%–6%, improved longissimus muscle area by 6%–8%, enlarged heart size by 5%–8%, and reduced backfat thickness by 6%–12% [8, 9].

The importance of ZBED6-IGF2 axis in regulating the growth of skeletal muscle had been demonstrated in several animal models [1, 10, 11], but its role in IMF deposition remains largely unclear. For instance, the ZBED6 knock-out mouse model, and *IGF2* knock-in mouse model carrying the natural mutation of porcine *IGF2* showed higher growth rate, increased lean mass, heart and kidney weight, but no significant changes were observed for white adipose tissues [10]. The IGF2-edited Chinese Bama pigs containing different mutant alleles around the regulatory motif, showed the elimination of the binding of repressor ZBED6, which significantly increased the growth rate of both founder and F1 generation, but notably, it did not affect meat quality, including fat content [12]. Similarly, the ZBED6-knockout Chinese Bama pigs showed markedly higher lean mass and heavier internal organs, but significantly decreased intramuscular fat deposition [13]. Therefore, it would be interesting to investigate what effects on skeletal muscle growth and meat quality of indigenous pigs will be anticipated by precise single-base editing in the ZBED6 binding motif in *IGF2*.

Base editing technology evolved from clustered regularly interspaced short palindromic repeats (CRISPR)-CRISPR-associated protein 9 (Cas9) technologies (CRISPR-Cas9), can directly install point-mutations in genomic DNA without inducing a DNA double-strand break (DSB), thus does not invoke the non-homologous end-joining (NHEJ) DNA repair mechanisms, generating fewer unwanted insertions and deletions (Indels) than classic CRISPR-Cas9 [14]. In this study, the *IGF2*-intron3-C3071T mutation was introduced into porcine embryonic fibroblasts (PEFs) isolated from a male Liang Guang Small Spotted pig (also known as Guangdong Small-ear Spotted pig) using cytosine base editor 3 (CBE3) to abolish the binding of ZBED6, and gene edited founder pigs were successfully obtained by somatic cell nuclear transfer technology (SCNT). Phenotypic, histological, and molecular characterization of F1 generation pigs were carried out. The regulatory roles of edited *IGF2* allele on muscle growth and IMF deposition were identified through in vitro functional analysis.

## Methods

### Animals

Pigs were raised at the Lemin Research and Development farm of China's Guangdong Yihao Indigenous Pig Research Institute Co., Ltd. Feed thrice a day until 2 months of age, after which the feeding was restricted according to the standard of finishing stage. During the breeding process, the wild-type and edited pigs were guaranteed to live and feed in the same conditions. Two F0 homozygous boars (*IGF2*^*T/T*^) were mated with four wild type sows (*IGF2*^*C/C*^) to generate F1 heterozygous generations (*IGF2*^*C/T*^). Meanwhile, wild-type boars from the descendants of the boar mated with a sow to produce fetus for isolating the donor cells for gene editing, were mated with wild type sows to produce F1 wild type pigs as control. The sows used for mating all were siblings. Thirty-one pigs were used for slaughter determination (15 pigs at 270-day-old, 16 pigs at 370-day-old).

### Vector construction

Guide sequence for sgRNA targeting the ZBED6 binding motif in intron 3 of porcine *IGF2* were designed using the open-source tool, CHOPCHOP (http://chopchop.cbu.uib.no/). The BE3 (a fusion protein of deaminase Apobec-1, Cas9 (D10A) nickase, and Uracil Glycosylase Inhibitor (UGI)) coding sequence was digested from the pCMV-BE3 (Addgene #73021, Cambridge, MA, USA), and cloned into the pX458 (Addgene #48138, Cambridge, MA, USA) to replace the 3 × FLAG-Cas9 coding sequence to generate the pX458-BE3 plasmid, which contains the enhanced green fluorescent protein (EGFP) reporter. Oligos of gRNA targeting porcine *IGF2*-intron3-3071 were synthesized (Sangon Biotech, Shanghai, China) and cloned into the plasmid pX458-BE3 through *Bbs* I restriction site to create the plasmid pX458-BE3-gRNA.

### Cell culture and transfection

PEFs were isolated from the 35 d fetus of Liang Guang Small Spotted pigs as previously described [15]. 1 × 10^6^ cells were resuspended in 100 μL buffer R (Invitrogen, Carlsbad, CA, USA) containing 10 µg pX458-BE3-gRNA plasmid, and electroporated at 1,650 V for 10 ms in 3 pulses by using the Neon transfection system (Invitrogen, Carlsbad, CA, USA). Transfected cells then were seeded in a 6-well plate with 2 mL preheated DMEM medium in each well. 48 h after transfection, cells were harvested using 0.25% trypsin/EDTA (Gibco, Gaithersburg, MD, USA), and EGFP-positive single cells were collected by fluorescence-activated cell sorting (FACS) with the use of Aria II cell sorter (BD Biosciences, San Jose, CA, USA). EGFP-positive cells were used for further culturing or the isolation of genomic DNA. The single cell was seeded into 96-well plates with 100 μL preheated DMEM medium containing 20% fetal bovine serum (FBS) and 0.1% penicillin–streptomycin in each well by using Aria II cell sorter. After three weeks of culture, the single cell was expanded for genotyping by PCR and sequencing.

### Somatic cell nuclear transfer (SCNT) and embryo transfer

Pig ovaries were collected from a local abattoir and transported to the laboratory in 0.9% (wt/vol) NaCl at 30 °C to 37 °C within 2 h. The in vitro-matured oocytes were denuded of cumulus cells and enucleated, PEFs harboring the desired mutations were injected into perivitelline space to form a reconstructed couplet. Fusion and activation were carried out with a single DC pulse of 120 kV/cm for 30 ms using a BTX Electro Cell Manipulator 2001 (BTX, San Diego, CA, USA). Then, the reconstructed embryos were placed in a PZM-5 medium, incubated at 37 °C overnight, and transferred into the oviduct of surrogate sows in estrus pregnancy status, detected on d 28 after embryo transfer by B-ultrasound. Cloned piglets were delivered until the surrogate sow carried the pregnancy to term.

### ChIP assay

100–150 mg frozen *longissimus lumborum* muscle tissue sample was placed in 2 mL pre-cooled PBS with protease inhibitor cocktail (PIC), and then crushed at 4 °C in a tissue mill to prepare single cell suspension. 1 mL supernatant was transferred to freshly prepared 11.1% formaldehyde solution and cross-linked for 15 min, then glycine was added to the sample at a final concentration of 0.125 mol/L to quench the cross-linking for 5 min. We modified the method of truCHIP® Chromatin Shearing Kit (Covaris PN520154, Woburn, MA, USA) to obtain a better nuclear preparation, and chromatin was sheared with AFA Focused-ultrasonicator to obtain fragment size distributions of 200–400 bp. Chromatin extract was conjugated with G-protein magnetic beads (Cell Signaling Technology 9006, Boston, Massachusetts, USA), 10 μg ZBED6 antibody was added to control and experimental groups, the rabbit IgG was used as a negative control. The mixture was precipitated overnight at 4 °C by rotating immunization. Chromosomes were eluted from antibody/protein G beads and purified. The final binding efficiency was analyzed by qPCR. Primers used for ChIP-qPCR were listed in Table S1, Additional file 1.

### Carcass traits and meat quality measurements

Birth weight and weight gain every month during the finishing stage were recorded. Pigs were euthanized and slaughtered at 270 and 370 days of age and fasted for 24 h before slaughter to assess meat production performance. The internal organs, head and hoofs of each pig were removed and weighed, after which the carcass weight was measured. Carcass traits were determined by measuring vertical length, slant length and backfat thickness, dissecting and weighing bone, sebum, suet, and lean mass from the left carcass; loin muscle area from the right carcass (the cross-sectional area of *longissimus lumborum* between the third and fourth penultimate ribs was depicted with sulphate paper, and was finally calculated with ImageJ). Meat quality traits included: meat color and marbling were assessed with colorimetric cards (Pork® Official Color & Marbling Quality Standards); pH (2 h), shear force, and pressing loss were measured with pH meter (PH-STAR, Matthaus, Gemany), digital display muscle tenderness meter (C-LM3B, College of Engineering, Northeast Agricultural University, China) and meat pressure meter (Tenovo Meat-1, Beijing, China), respectively. 200 g *longissimus lumborum* muscle near the penultimate fourth rib was selected for determination of intramuscular fat with Soxhlet diethyl ether extraction method (JOYN-SXT-06, Shanghai, China).

### Blood index analysis

Blood samples were taken from WT and *IGF*^*C/T*^ pigs one week before slaughter, the contents of IGF2, IGF1 and insulin in liver and serum were measured by porcine IGF2 ELISA kit (MLBio, Shanghai Enzyme-Linked Biotechnology Co., Ltd., Shanghai, China). Blood routine test was measured by automatic blood cell analyzer (URIT-2981, Guilin, China). Blood biochemical indexes were measured by automatic biochemical analyzer (URIT-8021A, Guilin, China).

### Histological and immunohistochemical analysis

Paraffin section: After the *longissimus lumborum* (LL), tenderloin (*Psoas major*, PM), gastrocnemius (GAS), semitendinosus (ST), backfat (BF) and internal organs were fixed in 10% neutral formalin solution (CITOTEST #80011–0042, Jiangsu, China) for 48 h, the automatic vacuum dehydrator (Leica ASP300S, Wetzlar, Germany) was used for 17 h dehydration according to the settings, and the tissues were trimmed into 5 mm × 5 mm blocks for embedding (Leica HistoCore Arcadia, Wetzlar, Germany), then the wrapped paraffin tissue was cut into 5 mm thick sections by using paraffin slicing machine (Leica HistoCore Autocut, Wetzlar, Germany) for H&E staining. Frozen section: The *longissimus lumborum* fixed for 48 h was consecutively dehydrated with 15% and 30% sucrose solution for one day, respectively. After embedding with Tissue-Tek® O.C.T. Compound (Sakura 4583, CA, USA), the tissue blocks were cut into 10 mm thick sections by using freezing microtome (Leica CM1950, Wetzlar, Germany) for Oil red O staining. Immunohistochemical double staining: After antigen repair, the primary antibody MYHC2 (Abcam ab51263, Boston, Massachusetts, USA, 1:400) was incubated overnight at 4 °C, rewarmed at room temperature for 15 min the next day, and the secondary antibody working solution (Abcam, ab205719, 1:2,000) was incubated at 37 °C for 45 min, then the freshly prepared diaminobenzidine (DAB) was used to color fast-twitch myofibers with brown. The above work was repeated for the slow muscle counterstaining, with the concentration of the primary and the secondary antibody working solution being 1:400 (Abcam, ab234431), 1:2,000 (Abcam, ab205718), respectively. 3-amino-9-ethylcarbazole (AEC) was used to color slow-twitch myofibers with red. Finally, hematoxylin was stained, sealed and dried, examined by microscope and scanned digitally (Hamamatsu Co. #NanoZoomer S360, Bridgewater, NJ, USA). Three visual fields were observed for each sample, the percentage of different muscle fibers were evaluated by Adobe Photoshop software. The antibodies used in the experiment were listed in Table S2, Additional file 2.

### Porcine primary mesenchymal stem cells isolation and in vitro differentiation

*Longissimus lumborum* muscle tissue from 7-day-old female *IGF2*^*C/T*^ and WT piglets was freshly isolated, minced, and digested with 2 mg/mL collagen type I (Biofroxx, Einhausen, Germany) in DMEM/F12 (Gibco, Gaithersburg, MD, USA) without fetal bovine serum (FBS) (Gibco, Gaithersburg, MD, USA) in a shaker water bath for 120 min at 37 °C. The precipitation was collected by centrifugation and then digested with 5 mL of 0.25% trypsin (Gibco, Gaithersburg, MD, USA) for 10 min, with the amount of trypsin added depending on the precipitation. Mesenchymal stem cells (MSCs) derived from skeletal muscle were filtered through a 70-μm cell strainer, and plated in 10 mL of growth medium (DMEM/F12 + 20% FBS + 1% penicillin–streptomycin). All experiments were performed in P5 passage cells, the passage and culture conditions of WT and *IGF2*^*C/T*^ cells were consistent. Proliferation experiments: EdU (RiboBio, Guangzhou, China), Ki67 (Abcam, ab15580) and real-time proliferation experiments were performed after one passage. Myogenic differentiation: Myoblast progenitor cells were obtained by differential adhesion method as described [16]. After 2 h of cell adherent culture, the supernatant was removed and replaced in a new culture dish. When the cells were fused to 80%, myoblast differentiation was induced in DMEM/F12 supplemented with 2% horse serum. On d 4, MyHC (Abcam, ab51263) immunofluorescence staining was performed. Adipogenic differentiation: After 2 d of cell fusion to 100%, 0.5 mol/L 3-isobutyl-1-methylxanthine (IBMX), 1 mmol/L dexamethasone (Dex), 10 mg/mL insulin and 10 μmol/L Rosiglitazone were added into the growth medium to induce adipogenic differentiation. On d 6, Oil red O staining, and immunofluorescence co-staining of perilipin (Abcam, ab3526) and bodipy (Sigma-Aldrich, St. Louis, Missouri, USA) were performed to evaluate the fully differentiated adipocytes. The antibodies used in the experiment were listed in Table S2, Additional file 2.

### Real-time cell proliferation monitoring assay

Real-time cell proliferation monitoring assay was conducted with xCELLigence RTCA system (ACEA biosciences, CA, USA). With 8,000 cells as a base, the growing cells were cultured in E-Plate 16 for a specified time, and the cell proliferation index was recorded using RTCA software 2.0.

### Immunofluorescence

Primary cells or 3T3-L1 cells were cultured in 6-well or 24-well plates, fixed in 4% paraformaldehyde for 30 min, and then permeated in 0.5% Triton X-100 for 20 min. After blocking for half an hour (Beyotime P0102, Shanghai, China), cells were incubated with the primary antibodies overnight at 4 °C. On d 2, cells were washed in PBST thrice and incubated with appropriate fluorescently labeled secondary antibodies for 1 h at room temperature. After rinsing the cells with PBST, nuclei were counterstained with DAPI for 10 min (1:500 in PBS). Antibodies are listed in Table S2, Additional file 2. Images were obtained by fluorescence reverse microscopy (Nikon, Tokyo, Japan).

### Oil red O staining

Lipid accumulation in adipocytes was measured by Oil red O staining (ORO). Cells were fixed with 4% formaldehyde for 30 min, washed with PBS, and then incubated with ORO (Sigma-Aldrich; ORO:deionized water = 3:2) for 1 h at room temperature in the dark. After staining, cells were quickly washed twice with 40% alcohol and observed under a Nikon bright-field microscope (Nikon, Tokyo, Japan). Finally, ORO was collected from the cells with 100% isopropyl alcohol and absorbance was measured at 562 nm.

### RNA sequencing

Tissues were placed in RNA-keeper (Vazyme, Nanjing, China) directly after collection. Total RNA was extracted using Trizol reagent kit (Invitrogen, Carlsbad, CA, USA), and RNA quality and integrity was assessed on an Agilent 2100 Bioanalyzer (Agilent Technologies, Palo Alto, USA). According to the manufacturer’s instructions, cDNA libraries were constructed and sequenced using Illumina Novaseq6000 by Gene Denovo Biotechnology Co. (Guangzhou, China). Sequenced reads were mapped to the reference pig genome (Sscrofa 11.1, download from https://asia.ensembl.org/Sus_scrofa/Info/Index) using StringTie v1.3.1 with default parameters [17, 18]. For each transcription region, the fragment per kilobase of transcript per million mapped reads (FPKM) value were calculated to quantify its expression abundance and variations, using RSEM [19] software. RNAs differential expression analysis was performed by DESeq2 [20] software between two different groups (and by edgeR [21] between two samples). The genes with the parameter of false discovery rate (FDR) below 0.05 and absolute fold change ≥ 2 were considered differentially expressed genes (DEGs).

### Cell treatment of inhibitors and activators

Primary cells or 3T3-L1 cells were treated with MK2206 (APExBio A3010, Houston, Texas, USA) 10 μmol/L and AICAR (MedChemExpress HY-13417, Monmouth Junction, USA) 2 mmol/L for 48 h to evaluate the cell proliferation, the confluent cells were cultured in adipogenic medium supplemented with MK2206 2 μmol/L and AICAR 1 mmol/L for 6 d to evaluate adipogenic differentiation.

### Quantitative PCR

For gene expression level detection, total RNAs were extracted using Trizol reagent kit (Invitrogen, Carlsbad, CA, USA) from muscle tissues or cell samples. cDNA was reverse transcribed following the instructions of HiScript® III RT SuperMix for qPCR (+ gDNA wiper) (Vazyme R323, Nanjing, China). Quantitative PCR (qPCR) analysis was performed on QuantStudio 7 Flex (Applied Biosystems) using AceQ Universal SYBR qPCR Master Mix (Vazyme Q511-03, Nanjing, China). GAPDH was used as an internal control for normalization using the comparative Ct (ΔΔCt) method. Primers used for qPCR were listed in Table S1, Additional file 1.

### Western blotting

Protein extracts from muscle tissues or cell samples were obtained using RIPA lysis buffer (Beyotime P0013B, Shanghai, China) supplemented with protease inhibitor phenylmethanesulfonyl fluoride (PMSF, Beyotime ST056, Shanghai, China) and incubated on ice for 30 min. Collected supernatant after centrifuging at 12,000 r/min for 20 min at 4 °C. Protein concentration was determined with BCA protein assay kit (meilunbio MA0082, Dalian, China), based on it, different samples were adjusted to the same concentration with RIPA, then mixed with 5× loading buffer and boiled for 15 min at 100 °C to denature proteins. Total proteins were electrophoresed on 8% or 10% (w/v) SDS-PAGE and transferred to PVDF membranes (Bio-Rad, Hercules, CA, USA). After blocking with QuickBlock Western blocker for 15 to 30 min, the samples were incubated with primary antibodies overnight at 4 °C, followed by incubation with appropriate secondary antibodies. Blots were visualized with an enhanced chemiluminescence (ECL) detection kit (MIKX MK-S100, Shenzhen, China). Antibodies were listed in Table S2, Additional file 2.

### Statistical analysis

The sample sizes for phenotypic characterization were 3–8 per genotype. The statistical data included at least three biological replicates. Data were presented as mean ± SEM. Values were analyzed by student’s *t*-test, one-way or two-way ANOVA analysis for multiple comparisons, using GraphPad Prism version 9.0. The level of significance was indicated as follows: ^*^P < 0.05, ^**^P < 0.01, ^***^P < 0.001 and *P* ≥ 0.05 represents not significant (ns).

## Results

### Generation of IGF2 base edited pigs

**Fig. 1:**
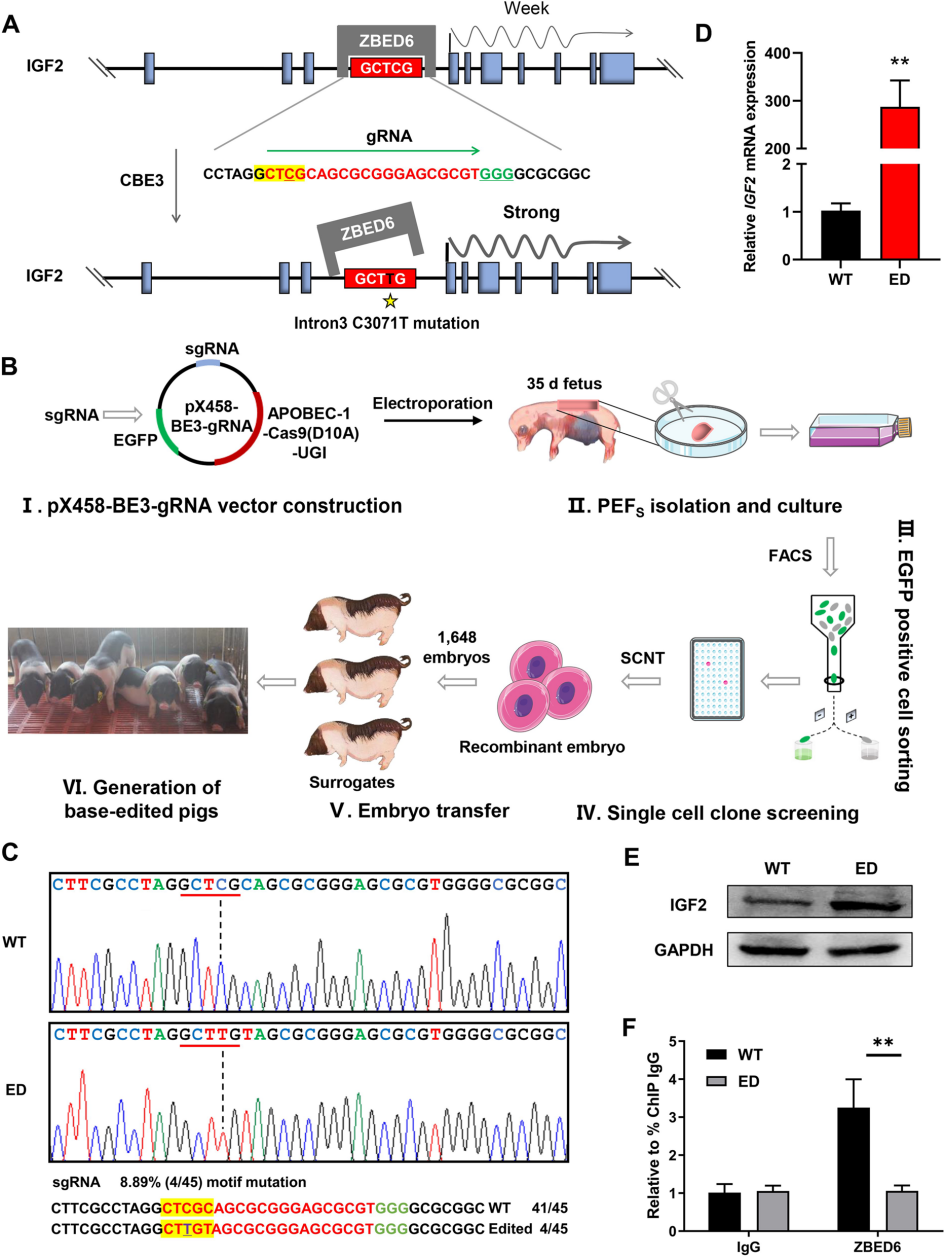
Generation of *IGF2*^*T/T*^ pigs using CBE3 editor. **A** Schematic diagram of the strategy to introduce C > T mutation in ZBED6 binding motif within intron 3 of porcine IGF2 by CBE3. The guide RNA sequence was shown in red, with the covering ZBED6 binding motif was boxed in yellow, and the C3071T was underlined. The PAM sequence was shown in green and underlined. **B** Schematic overview of the production of *IGF2*^*T/T*^ pigs. **C** The upper panel showed the representative chromatograms of DNA sequences of wild-type *IGF2* (WT) and *IGF2* with the introduced intron3-C3071T mutation (ED). The ZBED6 binding motif was underlined in red, and the *IGF2*-intron3-C3071T mutation site was indicated with black dashed. The lower panel showed the DNA sequences of the wild-type and mutant clones, with guide RNA recognition sites shown in red and the PAM sequence in green. The ZBED6 binding site motif is shown in a yellow box. **D** and **E** The mRNA expression and protein levels of IGF2 in PEFs. *n* = 3 per group. **F** The *longissimus lumborum* muscle tissue of 7-day-old *IGF2*^C/T^ and WT pigs were subjected for ChIP-qPCR analysis. *n* = 3 per group. All data were presented as mean ± SEM. ^*^P < 0.05, ^**^P < 0.01, student's *t*-test

To introduce a single base substitution in the ZBED6 binding motif within intron 3 of *IGF2*, we designed a gRNA to make the cytosines of the motif 5'-GCTCG-3' locate within the optimal editing window (16–20 nt upstream of protospacer adjacent motif, PAM) of CBE3 (Fig. 1A). The gRNA was cloned into base editor expression vector pX458-BE3 to generate pX458-BE3-gRNA, which carries an EGFP reporter (Fig. 1B and Fig. S1A). Then the pX458-BE3-gRNA plasmid was electroporated into PEFs. The EGFP-positive cells (account for 26.8%) (Fig. 1B and Fig. S1B) were sorted by fluorescence-activated cell sorting (FACS) to characterize the editing results by Sanger sequencing. Sequencing analysis revealed that the CBE3 was able to induce *IGF2*-intron3-C3071T mutation at a frequency of 8.89% (Fig. 1C). Interestingly, although *IGF2*-intron3-C3071T mutation frequency was below 10% in the sorted cell population, the *IGF2* transcriptional level increased by approximately 300-fold (Fig. 1D), and IGF2 protein level also increased apparently (Fig. 1E). Furthermore, we found myogenic genes including *MyoD*, *MyoG*, *DES* and pro-proliferative genes *Cyclin D1* were up-regulated significantly (Fig. S1C, Additional file 3), which suggest the introduced *IGF2*-intron3-C3071T mutation was able to effectively affect myogenesis process. We seeded single cell expressing EGFP into each well of a 96-well plate to obtain fifteen single cell clones. Fortunately, we identified one clone harbors the desired mutations from these clones, while other clones were intact. In this single cell clone, one allele of *IGF2* gene contains the desired *IGF2*-intron3-C3071T mutation, while the other allele contains the desired *IGF2*-intron3-C3071T mutation accompanied by an additional mutation (*IGF2*-intron3-C3073T) (single cell clone, SCC) (Fig. S1D, Additional file 3). This single cell clone was used as a donor for SCNT, and 1,648 recombinant embryos were transferred into the oviducts of 10 recipient surrogates. Finally, three recipient sows carried their pregnancies to term, resulting in the delivery of 22 piglets, in which 15 piglets were born alive and 11 piglets survived after 24 h (Table S3, Additional file 4). Unfortunately, only seven healthy piglets survived at 40 d (Fig. 1B), and four died early due to improper nursing or health problems.

Sanger sequencing of genomic DNA from cloned piglets showed that all piglets presented the genotype identical to the donor cell line (Fig. S1D). The *IGF2*-intron3-C3071T mutation significantly attenuated the binding ability of ZBED6 on the motif, as assessed by ChIP-qPCR in the muscle tissues (Fig. 1F). We have carried out off-target analysis (Table S4 and Fig. S2) and the integration of CBE3 expression plasmid in porcine genome (Fig. S3, Additional file 7) to exclude other effects on edited pigs. The additional *IGF2*-intron3-C3073T mutation outside of the ZBED6 binding motif did not further enhance the effect of *IGF2*-intron3-C3071T on pig growth (Fig. S4A, Additional file 8) and the expression of *IGF2* (Fig. S4B, Additional file 8). The intercross between two F0 homozygous boars (*IGF2*^*T/T*^) and four wild type sows (*IGF2*^*C/C*^) were established to generate paternal expression of the *IGF2*-intron3-C3071T mutation of the offspring (*IGF2*^*C/T*^). Therefore, the F1 heterozygotic individuals (*IGF2*^*C/T*^) were used for subsequent experiments. The following abbreviations were used in the figures to represent the two genotypes: Wild type (WT) = *IGF2*^*C/C*^ pigs; Edited heterozygote (ED) = *IGF2*^*C/T*^ pigs.

### *IGF2*^*C/T*^ pigs improved growth, carcass and meat quality

**Fig. 2:**
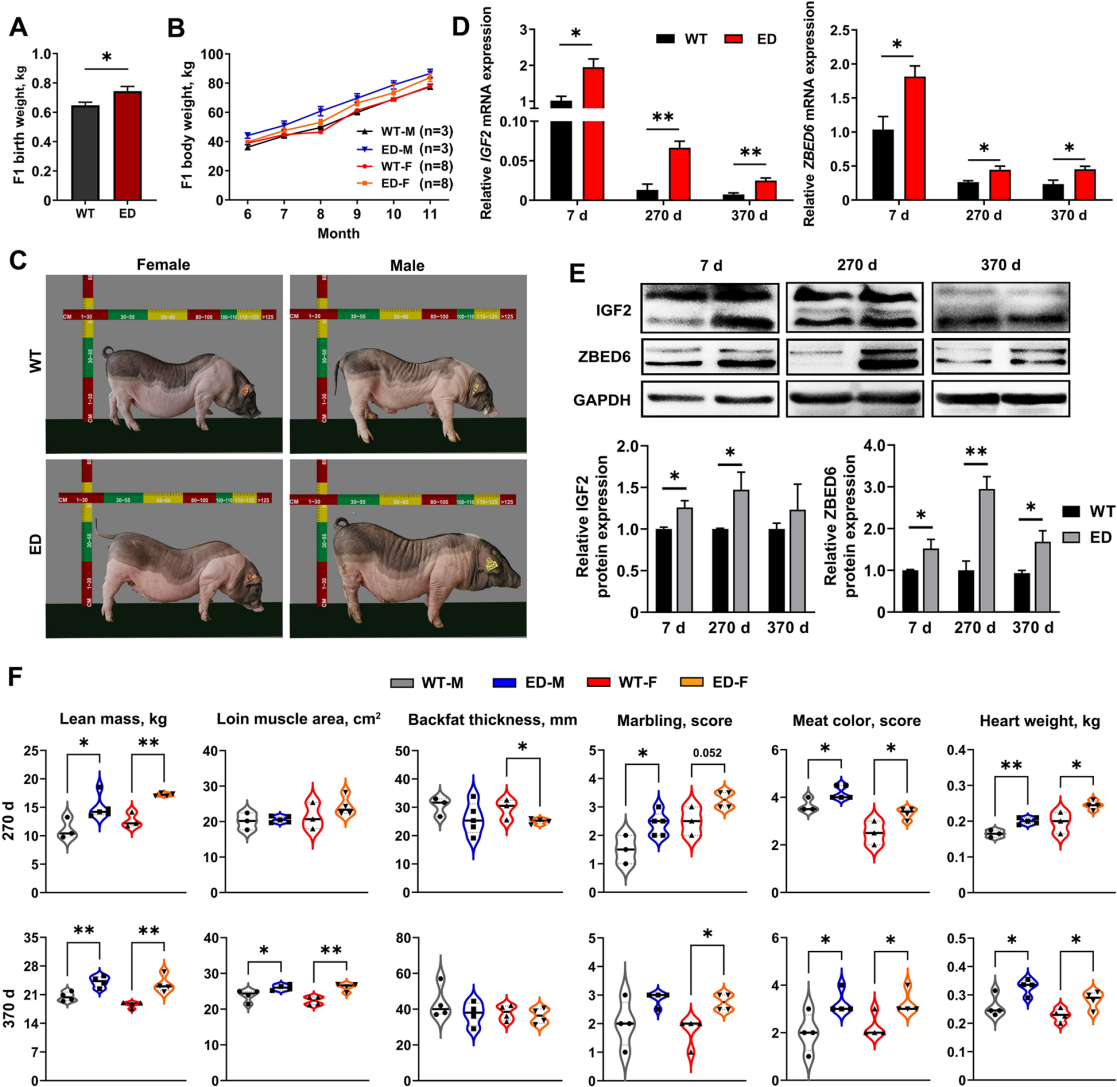
The improvement growth performance, carcass and quality traits of F1 *IGF2*^*C/T*^ generations. **A** The average birth weight of *IGF2*^*C/T*^ piglets were heavier than WT piglets regardless of genders (WT: *n* = 26; ED: *n* = 20). **B** Both male (M) and female (F) *IGF2*^*C/T*^ pigs have a better body weight gain than WT controls starting from 6 until 11 months of age. **C** Representative photos of WT and *IGF2*^*C/T*^ pigs at 5 months old. **D** and **E** mRNA and protein expression of IGF2 and ZBED6 in *longissimus lumborum* muscle of 7, 270 and 370-day-old female pigs. *n* = 3 per group. **F** Both male (M) and female (F) *IGF2*^*C/T*^ pigs had a better performance on carcass traits (lean mass, loin muscle area, backfat thickness), meat quality traits (marbling, meat color) and heart weight than the WT pigs of 270-day-old and 370-day-old. Each black point represented actual data (270 d M: WT, *n* = 3; ED, *n* = 5; 270 d F: WT, *n* = 3; ED, *n* = 4; 370 d M&F: *n* = 4 per group). All data were presented as mean ± SEM. ^*^P < 0.05, ^**^P < 0.01, student's *t*-test

The *IGF2*^*C/T*^ pigs were kept together with the wild-type littermates, and their body weight was measured monthly. As a result, the *IGF2*^*C/T*^ pigs had more heavier birth weight (Fig. 2A), and grew faster than the WT controls regardless of gender (Fig. 2B). Meanwhile, the improvement in growth rate was also evident by the increased body size of *IGF2*^*C/T*^ pigs, especially for male pigs (Fig. 2C and Fig. S4C). Quantitative PCR (qPCR) and Western blot were used to determine the IGF2 expression levels in *longissimus lumborum* (LL) muscle from 7, 270, and 370-day-old *IGF2*^*C/T*^ and WT pigs. As shown in Fig. 2D*, IGF2* mRNA was extensively expressed at younger age, and dramatically decreased at adult age*.* Compared with the WT controls, the mRNA and protein expression levels of *IGF2* were markedly increased in *IGF2*^*C/T*^ pigs, except for the protein level at 370-day-old. Meanwhile, both the mRNA and protein expression levels of *IGF2* repressor ZBED6 increased significantly of 7, 270 and 370-day-old *IGF2*^*C/T*^ pigs (Fig. 2D and E). Moreover, the content of IGF2, IGF1 (the principal mediator of growth hormone) and insulin increased apparently in liver and serum of *IGF2*^*C/T*^ pigs regardless of gender (Fig. S4D, Additional file 8), indicating the effective impact of *IGF2*-intron3-C3071T mutation on pig growth.

**Fig. 3:**
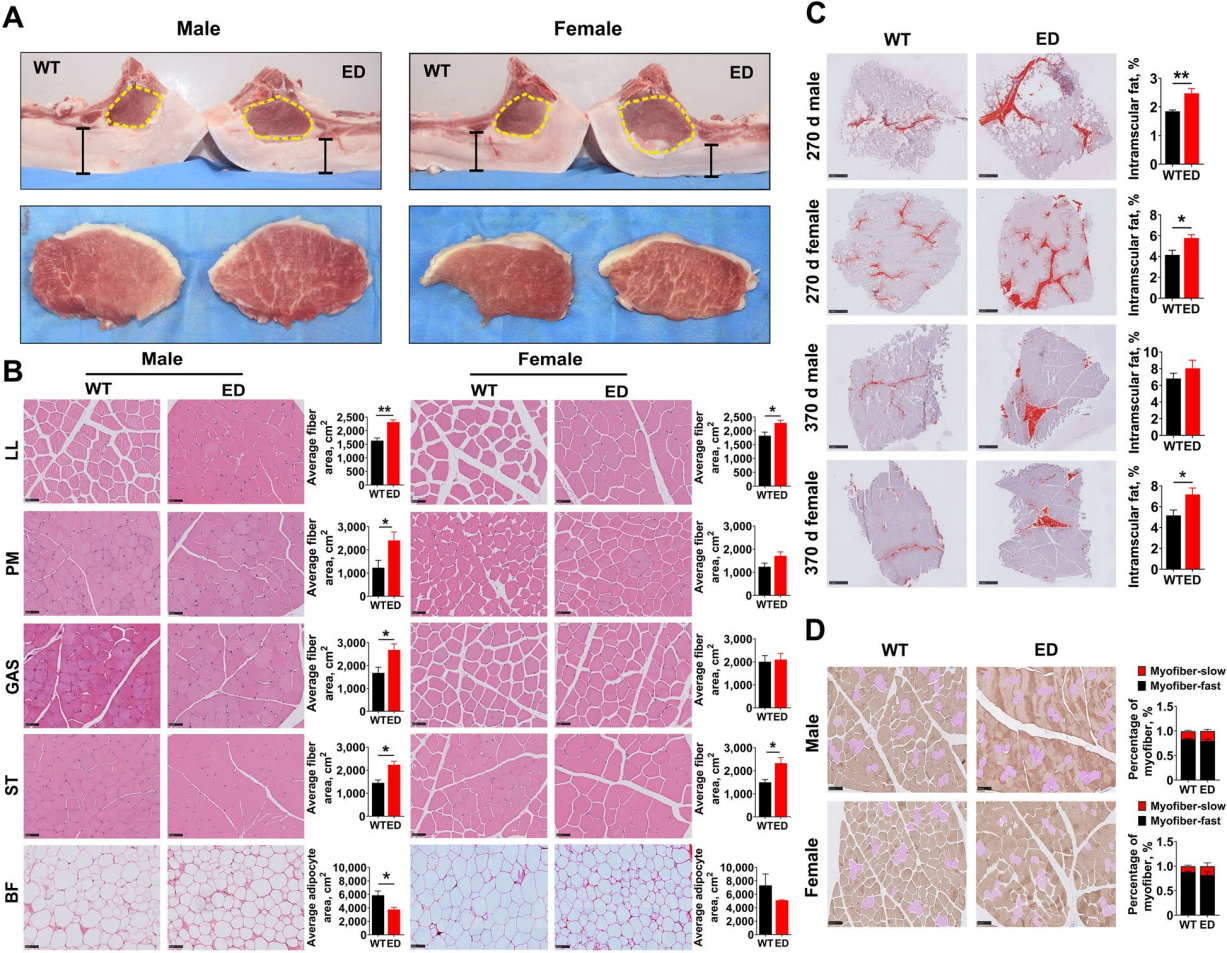
*IGF2*^*C/T*^ pigs showed muscle fiber hypertrophy and more IMF deposition. **A** Representative images of loin muscle area, backfat thickness and marbling of *IGF2*^*C/T*^ and WT pigs. The yellow dotted line circled loin muscle area and the black bar represented backfat thickness. **B** H&E staining of the *longissimus lumborum* (LL), *Psoas major* (PM), gastrocnemius (GAS), semitendinosus (ST) and backfat (BF) tissues of 270-day-old *IGF2*^*C/T*^ and WT pigs. The statistical analysis was presented as histogram in the right panel. Scale bar = 50 μm. *n* = 3 per group. **C** Frozen section: Oil red O staining in the LL muscle adipocytes of *IGF2*^*C/T*^ and WT pigs at 270 and 370-day-old. The IMF content was presented as histogram in the right panel. Scale bar = 1 mm. 270 d M: WT, *n* = 5; ED, *n* = 5; 270 d F: WT, *n* = 3; ED, *n* = 4; 370 d M&F: *n* = 3 per group. **D** Immunohistochemical double staining of fast-twitch and slow-twitch myofibers in the LL muscle of 270-day-old *IGF2*^*C/T*^ and WT pigs. The fast-twitch myofibers were stained with DAB in brown. The slow-twitch myofibers were stained with AEC in red. Scale bar = 50 μm. *n* = 3 per group. All data were presented as mean ± SEM. ^*^P < 0.05, ^**^P < 0.01, student's* t*-test

To further identify the meat performance and quality, the F1 generation carcass traits were measured at 270 and 370-day-old. Compared with WT pigs, greater lean mass (increased 18%–36%), larger loin muscle area (increased 3%–17%) (Fig. 3A), thinner backfat thickness (decreased 5%–16%) (Fig. 3A) were observed in *IGF2*^*C/T*^ pigs. Other carcass traits like carcass weight and slant length were improved significantly in 270-day-old *IGF2*^*C/T*^ pigs regardless of gender (Tables S5 and S6). Surprisingly, in addition to the effect on improving carcass quality, the *IGF2*^*C/T*^ pigs were able to improve meat quality, as reflected by improved marbling score (enhanced 0.75–1 score) and meat color score (enhanced 0.53–1.25 score) (Fig. 2F, Tables S5 and S6). Improvement on other meat quality parameters like pressing loss and pH in *IGF2*^*C/T*^ pigs were also observed (Tables S5 and S6). Additionally, the weight of internal organs (heart and kidney) was higher in *IGF2*^*C/T*^ pigs regardless of age, as reflected by heavier weight features (Table S5, Additional file 9) and (Table S6, Additional file 10). In general, the *IGF2*-intron3-C3071T mutation was able to improve both carcass and meat quality.

### *IGF2*^*C/T*^ pigs showed muscle fiber hypertrophy and more intramuscular fat deposition

The number of muscle fibers was already determined during the embryonic period [22], therefore, we suspected the increased lean mass in *IGF2*^*C/T*^ pigs should be due to the hypertrophy of muscle fibers. Histological examination exhibited an apparent increase of the cross-sectional area of muscle fibers in almost any examined muscle parts from *IGF2*^*C/T*^ pigs regardless of gender, including the *longissimus lumborum* (LL), tenderloin (*Psoas major*, PM), gastrocnemius (GAS), and semitendinosus (ST), except for the PM and GAS in female *IGF2*^*C/T*^ pigs. (Fig. 3B). Similarly, the thinner backfat thickness (BF) was due to the reduced size of adipocytes (Fig. 3B).

In different to the previous findings on the negative effect of the natural mutation *IGF2*-intron3-G3072A on meat quality traits, especially the IMF content in Western commercial pig breeds [1], we found the *IGF2*^*C/T*^ pigs had positive effects on meat quality traits, including improved marbling score, meat color score (Fig. 2F), and increased IMF content at both 270 and 370-day-old (Fig. 3C). The improved meat color has been proven to be correlated with muscle fiber type composition [23]. As a result, there was only a slight increase (not significant) in the percentage of slow-twitch muscle fiber in *IGF2*^*C/T*^ pigs (Fig. 3D), suggesting other factors affecting meat color should not be excluded.

To detect whether *IGF2*-intron3-C3071T mutation had adverse effects on pig health, we carried out blood routine test and blood biochemical test, and found that the measured parameters in *IGF2*^*C/T*^ pigs were grossly identical to the WT controls (Tables S7 and S8). Furthermore, the histological analysis revealed that no apparent pathological changes in heart, liver, spleen, lung, and kidney tissues of *IGF2*^*C/T*^ pigs (Fig. S4E, Additional file 8). These results indicated the *IGF2*-intron3-C3071T mutation did not affect pig health.

### Identification of co-expression networks associated with intramuscular fat by WGCNA

To identify genes associated with increased IMF, we performed transcriptome sequencing analysis of the LL muscle from *IGF2*^*C/T*^ and WT pigs at 270 and 370-day-old, and merged the different-age sequencing data with the IMF trait values for WGCNA (weighted gene co-expression network analysis) to explore the correlation between genes and a given trait. The selected gene sets were filtered prior to the WGCNA module analysis. The minimum power value (*R*^2^ = 0.8) when the scale independence reached a plateau was used as a parameter for subsequent analysis, and the changes in the average connectivity of genes under different power values were counted (Fig. S5A and B, Additional file 13). A gene clustering tree was constructed according to the correlation of expression levels between genes, and gene modules were divided by the clustering relationship (Fig. S5C, Additional file 13). Accordingly, a total of 19 WGCNA modules were identified, including 1 to 4,160 genes in each WGCNA module (Fig. S5D and E, Additional file 13). The expression patterns of the module genes in each sample were displayed with the module feature values (Fig. S5F, Additional file 13).

**Fig. 4:**
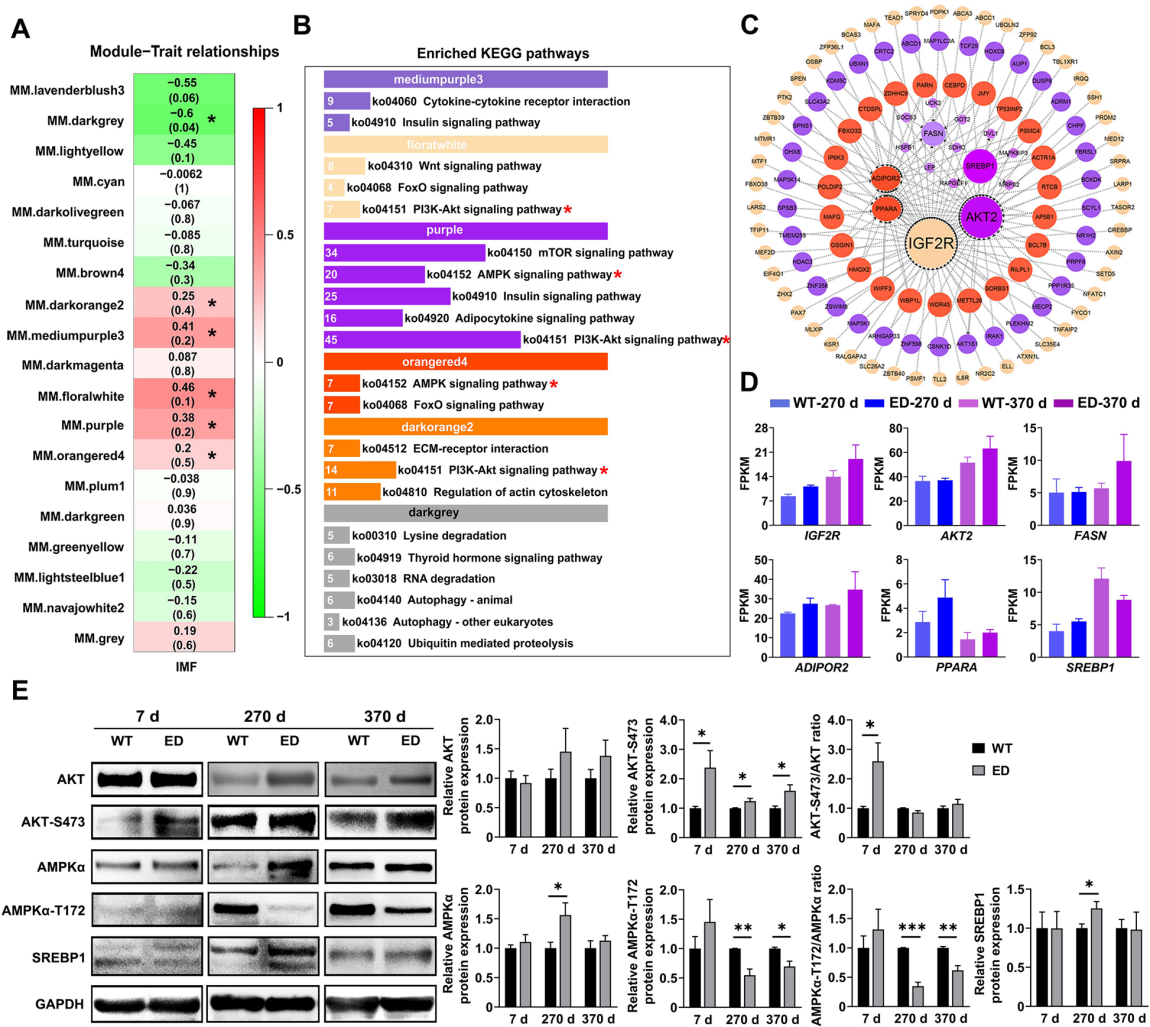
Identification and validation of co-expression networks associated with IMF content by WGCNA. **A** The 19 modules correlated to IMF content were identified by WGCNA. Values in each module represented the correlation coefficient between the module and the IMF content in the LL muscle of 270 and 370-day-old pigs. Red and green color noted the positive and negative correlation, respectively. The *P* values in parentheses represented the significance of the correlation between modules and IMF content. The six modules marked by asterisks were selected for further analysis. **B** Enrichment of the KEGG pathways positively or negatively associated with IMF content in the six selected modules from **A**. The number ahead of the items indicated the enriched gene number in each pathway. The marked asterisks represented the most enriched pathways. **C** Analysis of the interaction network of differentially expressed genes (DEGs) positively regulating IMF using Cytoscape software. The larger the node, the greater number of connections it has. Nodes come from each module in **B** shared the same color with the module. **D** Expression level of hub genes in the interaction network as measured by fragments per kilobase per million (FPKM). **E** Western blot analyzed the protein levels of AKT, AKT-S473, AMPKα, AMPKα-T172 and SREBP1. *n* = 3–4 per group. All data were presented as mean ± SEM. ^*^P < 0.05, ^**^P < 0.01, student's* t*-test

We focused on five modules (floratwhite, mediumpurple3, purple, darkorange2 and orangered4) positively correlated with and one module (darkgrey) negatively correlated with IMF (Fig. 4A). The five positively correlated modules were mainly enriched in insulin signaling pathway, PI3K-AKT signaling pathway and AMPK signaling pathway, while the negatively correlated module was mainly enriched in RNA degradation, autophagy-animal and ubiquitin mediated proteolysis pathway, etc. (Fig. 4B). These data suggested that positively correlated modules and genes may play a key role in IMF deposition during the growth from 270 to 370 d. We further mined the hub genes among the five focused modules through gene interaction network analysis (Fig. 4C), and identified *IGF2R*, *AKT2*, *FASN*, *ADIPOR2*, *PPARA* and *SREBP1* (genes associated with adipogenesis) were up-regulated in *IGF2*^*C/T*^ pigs, excepting the down-regulation of SREBP1 in 370-day-old *IGF2*^*C/T*^ pigs (Fig. 4D).

### *IGF2*^*C/T*^ pigs regulated IMF deposition through PI3K-AKT/AMPK signaling pathway

Increasing evidence has proved that activation of the phosphatidylinositol 3-kinase and AKT (PI3K-AKT) pathway can induce skeletal muscle hypertrophy [24]. AMP-activated protein kinase (AMPK), as an energy sensor, stimulating fatty acid oxidation and inhibiting the production of cholesterol and triglycerides in adipose cells, has been proven to be inhibited by AKT [25]. Here, we found a slight up-regulation of AKT proteins in 270 and 370-day-old *IGF2*^*C/T*^ pigs, and significantly increased AKT-S473 phosphorylation in 7, 270 and 370-day-old *IGF2*^*C/T*^ pigs (Fig. 4E), indicating enhanced PI3K-AKT signaling happened in the early age of edited pigs. In contrast, significantly decreased AMPKα-T172 phosphorylation was observed in 270 and 370-day-old *IGF2*^*C/T*^ pigs (Fig. 4E), suggesting attenuated AMPK signaling in adult edited pigs.

Given the promoted IMF deposition and the inhibition of AMPK signaling in the edited pigs, we examined the expression of related genes to evaluate adipogenic potential. qPCR analysis revealed peroxisome proliferator-activated receptor-gamma (*PPAR*G), one of the master regulators of adipocyte differentiation, increased significantly in 7 and 270-day-old *IGF2*^*C/T*^ pigs; The sterol regulatory element-binding protein 1 (*SREBP1*), a master regulator of lipogenic gene expression, also increased significantly in 270-day-old *IGF2*^*C/T*^ pigs; The acetyl-CoA carboxylase (*ACC*), the rate-limiting enzyme in fatty acid synthesis, increased dramatically in 7, 270 and 370-day-old *IGF2*^*C/T*^ pigs; While only hydroxymethylglutaryl-CoA (*HMG-CoA*) reductase, the rate-limiting enzyme of cholesterol biosynthesis, no significant changes were observed between *IGF2*^*C/T*^ and WT pigs (Fig. S6, Additional file 14). Furthermore, the significantly increased protein level of SREBP1 was confirmed in 270-day-old *IGF2*^*C/T*^ pigs (Fig. 4E). These results indicated that IGF2 regulated intramuscular adipogenesis by inhibiting AMPK signaling through the activation of PI3K-AKT pathway.

### *IGF2*-intron3-C3071T mutation promoted in vitro cell proliferation, myogenesis and adipogenesis

**Fig. 5:**
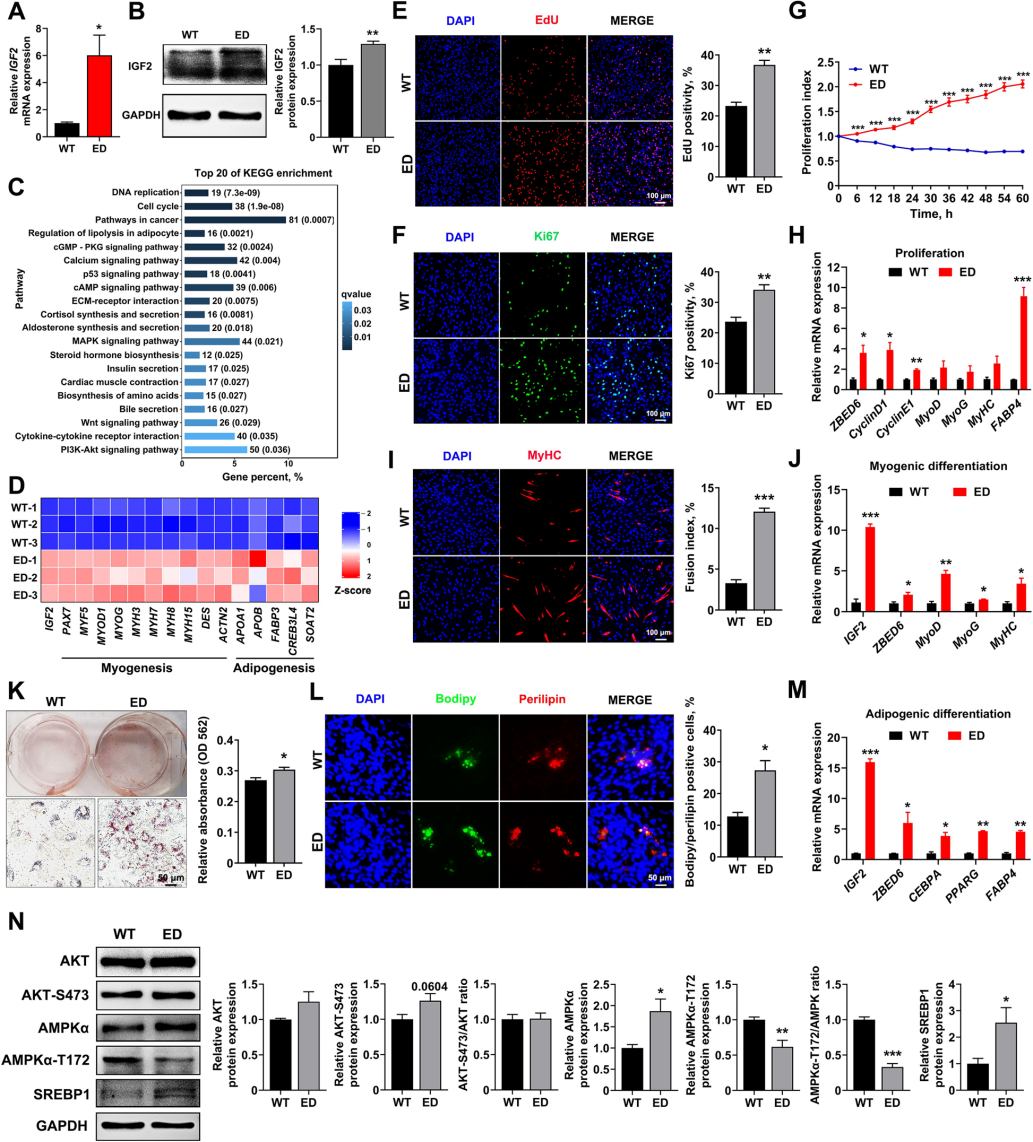
*IGF2*-intron3-C3071T mutation promoted cell proliferation, myogenesis and adipogenesis of MSCs isolated from the LL muscle of 7-day-old female piglets. **A** and **B** The mRNA expression and protein levels of IGF2 were detected in MSCs. *n* = 3 per group. **C** The top 20 of KEGG pathways enriched by transcriptomic analysis in MSCs. **D** Heatmaps of *IGF2*, and key genes involved in myogenesis and adipogenesis. **E** and **F** EdU and Ki67 immunofluorescent staining were performed between *IGF2*^*C/T*^ and WT MSCs, the percentage of EdU-positive or Ki67-positive cells were presented as histogram in the right panel. Scale bar = 100 μm. *n* = 3 per group. **G** Real-time cell proliferation monitoring assay of the proliferation index between *IGF2*^*C/T*^ and WT MSCs. *n* = 8 per group. **H** qPCR analyzed the mRNA levels of related genes in the proliferation phase. *n* = 3 per group. **I** Immunofluorescent staining of MyHC was performed to detect myotube formation at d 4 of induced myogenic differentiation. The fusion index was presented as histogram in the right panel. Scale bar = 100 μm. *n* = 3 per group. **J** qPCR analyzed the mRNA levels of related genes at d 4 of induced myogenic differentiation. *n* = 3 per group. **K** Oil red O staining of lipid droplet formation at d 6 of induced adipogenic differentiation. The absorption value of triglyceride content was presented as histogram in the right panel. Scale bar = 50 μm. *n* = 3 per group. **L** Immunofluorescence co-staining of perilipin and bodipy between *IGF2*^*C/T*^ and WT MSCs at d 6 of induced adipogenic differentiation. Scale bar = 50 μm. *n* = 3 per group. **M** qPCR analyzed the mRNA levels of related genes at d 6 of adipogenic differentiation. *n* = 3 per group. **N** Western blot analyzed the protein levels of AKT, AKT-S473, AMPKα, AMPKα-T172 and SREBP1 at d 6 of induced adipogenic differentiation. All data were presented as mean ± SEM. ^*^P < 0.05, ^**^P < 0. 01, ^***^P < 0.001, student's *t*-test

We further carried out in vitro studies to validate the regulatory role of *IGF2*-intron3-C3071T mutation (*IGF2*^*C/T*^) on porcine myogenesis and adipogenesis. The mesenchymal stem cells (MSCs) were isolated from LL muscle of 7-day-old *IGF2*^*C/T*^ and WT female piglets. In accordance with the findings in vivo, the mRNA and protein levels of IGF2 was significantly increased in *IGF2*^*C/T*^ MSCs (Fig. 5A and B). The results of transcriptomic analysis revealed that *IGF2*-intron3-C3071T mutation affected pathways involved in proliferation, myogenesis, and adipogenesis, etc. (Fig. 5C), as confirmed by the significantly enriched DEGs associated with them (Fig. 5D), implying this mutation could enhance myogenesis and adipogenesis.

In parallel, both EdU and Ki67 staining assays, and the real-time proliferation analysis demonstrated that *IGF2*-intron3-C3071T mutation significantly promoted MSCs proliferation (Fig. 5E–G). Meanwhile, qPCR analysis showed the increased pro-proliferative genes (*Cyclin D1* and *Cyclin E1*), myogenic genes (*MyoD*, *MyoG* and *MyHC*) and adipogenic genes (*FABP4*) were statistically significant or near significant in *IGF2*^*C/T*^ MSCs (Fig. 5H). These results confirmed the major findings in transcriptomic analysis. Of note, the mRNA expression of *IGF2* and its repressor ZBED6 was markedly increased in *IGF2*^*C/T*^ MSCs (Fig. 5H), which was consistent with the in vivo findings (Fig. 2D), implying elevated IGF2 expression may have a feedback regulatory role on ZBED6 expression.

In addition, the differentiation of *IGF2*^*C/T*^ myoblasts resulted in the formation of more multinucleated myotubes with larger diameter post 4 d of induced differentiation (Fig. 5I), accompanied by increased expression of myogenic genes including *MyoD*, *MyoG* and *MyHC* (Fig. 5J). Meanwhile, Oil Red O staining revealed that *IGF2*^*C/T*^ MSCs increased triglyceride content with the large number of lipid droplets post 6 d of induced adipogenic differentiation (Fig. 5K), in accordance with the immunofluorescent co-staining of bodipy and perilipin (Fig. 5L), accompanied by increased expression of adipogenic genes including *C/EBPA*, *PPARG*, and *FABP4* (Fig. 5M).

### IGF2 regulated adipogenic differentiation via PI3K-AKT/AMPK pathway

Further, we found *IGF2*^*C/T*^ MSCs enhanced PI3K-AKT signaling and inhibited AMPK signaling, reflected by increased level of phosphorylated AKT and significantly decreased level of phosphorylated AMPKα of induced adipogenic differentiation (Fig. 5N). Consistently, *IGF2*^*C/T*^ MSCs significantly increased the expression of master adipogenic regulator SREBP1 (Fig. 5N), indicating SREBP1 may be an effective target of IGF2 in promoting adipogenesis. These results were in agreement with in vivo findings (Fig. 4E).

**Fig. 6:**
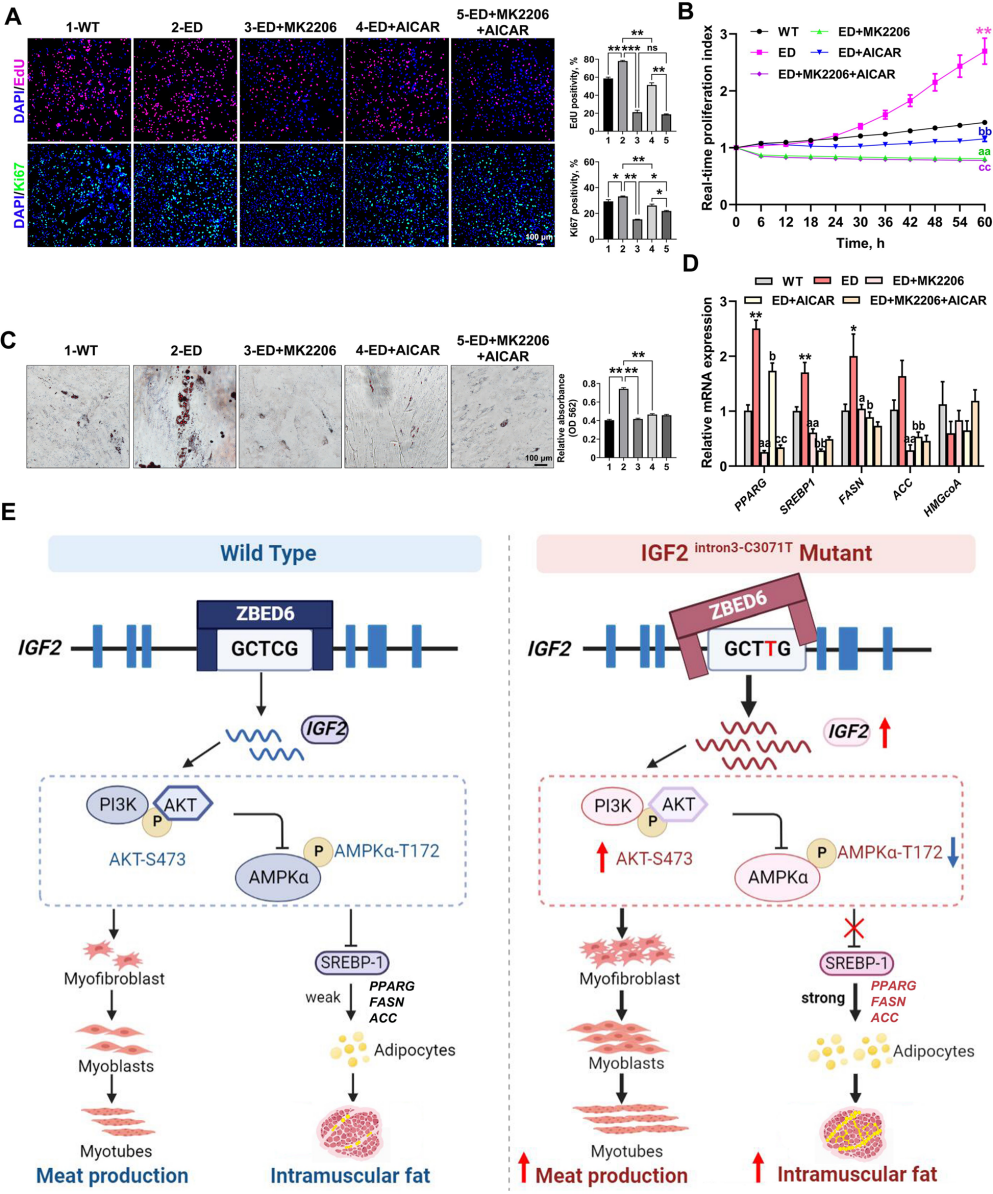
*IGF2*-intron3-C3071T mutation improved adipogenesis through PI3K-AKT/AMPK pathway. **A** EdU and Ki67 immunofluorescent staining were performed between WT MSCs, ED MSCs, and ED MSCs with the treat of MK2206, AICAR, or MK2206 + AICAR. The percentage of EdU-positive or Ki67-positive cells were presented as histogram in the right panel. Scale bar = 100 μm. **B** Real-time cell proliferation monitoring assay was carried to measure the proliferation index of the above 5 groups, *n* = 3 per group. Mark of significance, *: WT vs. ED; a: ED vs. ED + MK2206; b: ED vs. ED + AICAR; c: ED + AICAR vs. ED + MK2206 + AICAR. Significance (*P* < 0.01) was reached from 30 to 60 h, only 60 h was marked in the figure. **C** Oil red O staining of lipid droplet formation at d 6 of induced adipogenic differentiation in the above 5 groups. The absorption value of triglyceride content was presented as histogram in the right panel. Scale bar = 100 μm. **D** qPCR analyzed the relative transcription levels of downstream target genes in AMPK signaling pathway. Mark of significance was same as **B**. **E** Schematic diagram of the regulatory mechanism of *IGF2*-intron3-C3071T mutation on improved myogenesis and adipogenesis. All data were presented as mean ± SEM. *n* = 3–4 per group. ^*^P < 0.05, ^**^P < 0.01, ^***^P < 0.01, ns: *P* > 0.05, one-way ANOVA or two-way ANOVA

To determine whether the effect of *IGF2*-intron3-C3071T mutation on MSCs proliferation and adipogenic differentiation was mediated by PI3K-AKT/AMPK signaling, the highly specific AKT inhibitor MK2206 and AMPK signaling activator AICAR, were applied to disrupt the according signaling functions. As a result, both EdU and Ki67 staining assays (Fig. 6A), and the real-time proliferation analysis (Fig. 6B) revealed that the effect of *IGF2*-intron3-C3071T mutation on MSCs proliferation was potently attenuated (PI3K-AKT) and less attenuated (AMPK) by inhibiting the PI3K-AKT signal pathway and activating the AMPK signal pathway, respectively. The treatment combination of these two signal pathways did not seem to present an additive effect (Fig. 6A and B), suggesting the influence of *IGF2*-intron3-C3071T mutation on MSCs proliferation was mainly mediated by PI3K-AKT signal pathway.

Inhibiting PI3K-AKT signal pathway or activating AMPK signal pathway individually potently attenuated the effect of *IGF2*-intron3-C3071T mutation on MSCs adipogenesis, as evidenced by dramatically reduced droplet formation (Fig. 6C), and significantly decreased the mRNA expression of AMPK downstream targets *SREBP1*, *PPARG*, *ACC* and *FASN*, which are involved in lipid metabolism and synthesis (Fig. 6D). However, the disruption of either PI3K-AKT or AMPK signal pathway did not significantly affect *HMGCoA* expression (Fig. 6D), indicating PI3K-AKT/AMPK signaling may not participate in cholesterol synthesis in our model.

Similar results of IGF2-PI3K-AKT/AMPK axle in cell proliferation and adipogenesis were confirmed in 3T3-L1 preadipocytes through overexpressing porcine *IGF2* (OE IGF2) (Fig. S7, Additional file 15). Taken together, these data supported the notion that PI3K-AKT/AMPK signal pathway was involved in the accelerated cell proliferation and enhanced adipogenesis induced by *IGF2*-intron3-C3071T mutation.

## Discussion

The domestic pig is an important livestock raised worldwide to provide meat for human consumption [26]. Selective breeding has been successfully applied to enhance meat production in Western pig breeds, while the progression rate is often slow and limited to variants that exist within the breeding population [27]. In recent years, CRISPR-Cas9 has been used to create site specific changes to the genomes of livestock to improve important economic traits in a single generation [28], but the HDR dependent point mutation induced by CRISPR-Cas9 often induces additional (bystanding) indels, which may confound the genetic effects of target mutations [29]. Therefore, precise single base substitution provides a strategy for accurate genetic improvement in pigs [30]. The recently developed base editors including cytidine base editors (CBEs) and adenine base editors (ABEs) could enable direct generation of precise point mutations in genomic DNA without generating DSBs and HDR, resulting in minimum indels formation [31]. In this study, CBE3 was used to induce a base substitution (*IGF2* intron3-C3071T) into LGSS genome, and improved both meat production and meat quality much faster than the selective breeding. We found there was no additional indel occurred around the target site of gRNA both in edited cells and pig individuals. However, CBE3 was able to induce another C to T substitution in the editing window (16–20 nt upstream of PAM), but this additional single base mutation did not affect the main economic traits. In past decades, the breeding goals of reducing backfat thickness and increasing lean mass have been achieved through selective breeding, meanwhile, it also led to a decrease in IMF, which did not satisfy consumers on the demand for high-quality meat [32]. Our study first overcame the dilemma between meat production and quality in pig breeding through precise base editing by using CBE3.

The natural occurred single base mutation (*IGF2*-intron3-G3072A) within the ZBED6 binding motif 5'-GCTCG-3' was able to release the repression of ZBED6 on *IGF2* expression, leading to the up-regulation of *IGF2*, resulting in increased lean mass and reduced backfat thickness [6, 8]. Whether any other base mutation in the ZBED6 binding motif has the same genetic effect has not yet been verified. Our study demonstrated that the precise mutation of the second cytidine (*IGF2* intron3-C3071T) in the binding motif was also able to release the repression of ZBED6, and led to significantly increased lean production and reduced backfat in *IGF2*^*C/T*^ pigs, which indicated the previously identified IGF2-ZBED6 binding motif was crucial, any mutation of five bases was possible to release the inhibition of ZBED6 on *IGF2* transcription, and presenting significant genetic effects on porcine lean mass and backfat thickness. Interestingly, the *IGF2*-intron3-C3071T mutation may have a negative feedback effect on the expression of ZBED6, as significantly increased expression of ZBED6 was discovered in *IGF2*^*C/T*^ pigs with increased expression of IGF2. To our knowledge, this phenomenon has been never reported. Therefore, whether the *IGF2* signaling participated in the regulation of its repressor ZBED6 expression is an interesting topic that deserves further investigation in future.

Compared with the ~ 4% increase in muscle growth in Western pigs carrying the natural *IGF2*-intron3-G3072A (*IGF2*^*G/A*^) mutation [8], the increased muscle growth (18%–36%) in our *IGF2*^*C/T*^ pigs was more pronounced. Varying degrees of positive effects on muscle growth were also observed in other mouse and pig models [10, 12]. This difference is possibly due to the long history extensive selection for lean growth in Western pigs, resulting in the accumulation of various genetic mutations controlling muscle growth, which may mask the promotive effect of natural *IGF2*^*G/A*^ mutation on muscle growth. Another striking difference was that the natural *IGF2*^*G/A*^ mutation did not affect serum IGF2 concentration in Western pigs [8], whereas significantly higher concentrations of IGF2 in both liver and serum were observed in *IGF2*^*C/T*^ pigs, accompanied by elevated concentrations of IGF1 and insulin. This could also explain why *IGF2*^*C/T*^ pigs showed a significant effect on growth than Western pigs, cause the circulating IGF2, IGF1 and insulin were all able to stimulate muscle growth [33, 34]. The most intriguing difference was that meat quality parameters including meat color, marbling, water retention, and especially IMF content were improved in *IGF2*^*C/T*^ pigs. We suspect the molecular regulatory mechanism of IMF deposition in LGSS may be different to that in Western pig breeds, making this breed more sensitive to the enhanced IGF2 expression induced by *IGF2*-intron3-C3071T mutation. Moreover, this improvement of meat quality was not found in *IGF2*-intron3-deletion pigs [12] and the ZBED6-knockout pigs [13], which showed markedly higher lean mass, but no significant difference in meat quality or significantly decreased IMF deposition, we suspect the different may come from that they measured meat quality at an earlier stage (6 or 8 months of age) than us (270 and 370 d). Also, whether a different molecular regulatory mechanism of IMF deposition exists between LGSS and Bama pigs may account for this should not be excluded. Therefore, our study first presented an example for simultaneous improvement of both meat production and quality in pig breeding through single base editing.

The strikingly more IMF observed in the muscle of *IGF2*^*C/T*^ pigs was speculated to be due to enhanced proliferation of adipogenic progenitors, as in vitro cultured *IGF2*^*C/T*^ MSCs presented much stronger proliferative capacity. Both myogenic and adipogenic progenitors are derived from MSCs, and a fraction of MSCs were called fibro-adipogenic progenitors (FAPs) [35]. Enhanced proliferative capacity of FAPs has been proven to contribute to increase adipogenesis of Japanese Wagyu skeletal muscles, resulting in higher marbling content [35, 36]. Therefore, like the Japanese Wagyu beef, the higher marbling content in *IGF2*^*C/T*^ pigs could be attributed to the enhanced proliferative capacity of adipogenic progenitors. As the total number of adipocytes in skeletal muscle was mainly determined during the fetal and early postnatal stages, the notably increased IMF deposition from about 250-day-old during the fattening stage was due to enhanced adipogenesis [35]. In consequence, the significantly up-regulated expression of adipogenic transcription factor SREBP1 was in the muscle of 270-day-old but not 370-day-old *IGF2*^*C/T*^ pigs, implying that the enhanced in vivo maturation of adipocytes in *IGF2*^*C/T*^ pigs may concentrate in a short period around 250-day-old. The higher adipogenic differentiation capability of *IGF2*^*C/T*^ MSCs in vitro also supported an enhanced adipogenesis that existed during muscular development of *IGF2*^*C/T*^ pigs.

Transcriptome-based WGCNA identified the involvement of PI3K-AKT and AMPK signaling pathways in the regulation of improved IMF deposition in *IGF2*^*C/T*^ pigs. Increasing studies have proven *IGF2* can promote cell proliferation and differentiation through the PI3K-AKT pathway [5], while the energy and nutrient sensor AMPK plays an opposite role in governing cell growth [37]. It has been well established that PI3K-AKT negatively regulates the AMPK signaling pathway [25]. Potent activation of PI3K-AKT pathway in the muscle of 7-day-old *IGF2*^*C/T*^ pigs and in vitro induced adipogenic differentiation of MSCs, together with the significantly inhibited AMPK signaling in MSCs, implied an enhanced proliferation of adipogenic progenitors, which may contribute to the formation of more adipocytes. More importantly, AMPK plays a crucial role in anti-regulating adipogenesis [38], and it has been proven AMPK can suppress SREBP1, a nuclear transcription factor that regulates the synthesis of fatty acid and cholesterol [39], through inhibiting its proteolytic processing and repressing its target gene expression, leading to reduced adipogenesis and lipid accumulation [40]. Significantly increased expression of SREBP1 in the muscle of 270-day-old *IGF2*^*C/T*^ pigs and in vitro induced adipogenic differentiation of MSCs, together with the enhanced expression of adipogenesis related genes such as *PPARG*, *FASN* and *ACC* in muscle or MSCs, suggesting the important role of SREBP1 in promoting IMF deposition in *IGF2*^*C/T*^ pigs. This was supported by the observation of remarkably decreased expression of SREBP1 and reduced adipogenesis in vitro induced adipogenic differentiation of MSCs with AMPK signal pathway manually activated. Moreover, IGF1 and insulin have been reported to induce the expression of SREBP1, leading to increased total lipid production [41]. The increased insulin and IGF1 content in serum of *IGF2*^*C/T*^ pigs may also contribute to the increased expression of SREBP1, leading to improved IMF deposition. To the best of our knowledge, we are the first to show the regulation of IMF deposition by *IGF2* through SREBP1 via the PI3K-AKT/AMPK signaling pathways.

The health status of *IGF2*^*C/T*^ pigs were evaluated by histological analysis, blood routine test and blood biochemistry test. In a whole, the *IGF2*^*C/T*^ pigs were physiologically normal, without obvious pathological conditions. However, increased red blood cell (RBC), reduced mean corpuscular volume (MCV), mean corpuscular hemoglobin (MCH) and cell distribution width-variable coefficient (RDW-CV) were observed in 270-day-old *IGF2*^*C/T*^ male pigs, suggesting the *IGF2*^*C/T*^ male pigs have more uniform but smaller red blood cells. Increased activation of PI3K-AKT pathway in erythroid precursor cells has been found to develop more RBCs [42]. Therefore, we speculated that increased expression of IGF2 in *IGF2*^*C/T*^ male pigs could enhance PI3K-AKT signaling, leading to increased proliferation of erythroid precursor cells, and generating more RBCs. We also noticed a significantly reduced glucose (GLU) level in serum of 270-day-old *IGF2*^*C/T*^ female pigs. It is reasonable that the more prominent increased level of insulin in both liver and serum of *IGF2*^*C/T*^ female pigs could stimulate more glucose uptake by cells, and reduce the glucose level in serum. This finding was in accordance with that found in *IGF2* knock-in mouse model [10]*.*

## Conclusions

This study has broken through the unfavorable genetic correlation between lean growth and pork quality for the first time through a precise single base substitution in the *IGF2* gene of a Chinese indigenous pig breed by using base editing technology. In this study, we successfully produced the single-base edited (*IGF2*-intron3-C3071T) Chinese Liang Guang Small Spotted pigs through CBE3 and SCNT. The created base edited pigs presented both improvement in lean production and pork quality. We demonstrated the regulation of IMF deposition by *IGF2* through SREBP1 via the PI3K-AKT/AMPK signaling pathways (Fig. 6E). Our study provides a further understanding of the biological functions of IGF2 and an example for improving porcine economic traits through precise base editing.

### Supplementary Information

**Additional file 1: Table S1.** List of primers used in this study.

**Additional file 2: Table S2.** Antibodies and application used in this study.

**Additional file 3: Fig. S1.** Evaluation of gRNA activity and characterization of mutations in single cell clone and edited pigs.

**Additional file 4: Table S3.** Summary of generation of *IGF2*^*T/T*^ pigs through SCNT.

**Additional file 5: Table S4.** Primer details for detection of off-target sites.

**Additional file 6: Fig. S2.** Off-targeting analysis by Sanger sequencing.

**Additional file 7: Fig. S3.** Detection of integration of pX458-BE3-gRNA vector in genome of gene-edited pigs.

**Additional file 8: Fig. S4.** Growth phenotype and health detection of *IGF2*^*C/T*^ F1 generation pigs.

**Additional file 9: Table S5.** Carcass traits and meat quality between WT and *IGF2*^*C/T*^ pigs at 270-day-old.

**Additional file 10: Table S6.** Carcass traits and meat quality between WT and *IGF2*^*C/T*^ pigs at 370-day-old.

**Additional file 11: Table S7.** Blood routine test of WT and *IGF2*^*C/T*^ pigs at 270-day-old.

**Additional file 12: Table S8.** Blood biochemistry test of WT and *IGF2*^*C/T*^ pigs at 270-day-old.

**Additional file 13: Fig. S5.** Identification of modules correlated to intramuscular fat content by weighted gene correlation network analysis (WGCNA).

**Additional file 14: Fig. S6.** qPCR analysis of the relative transcription levels of downstream target genes in AMPK signaling pathway.

**Additional file 15: Fig. S7.** Overexpression of IGF2 in 3T3-L1 cells promoted cell proliferation and adipogenic differentiation through the PI3K-AKT/AMPK pathway.

## Data Availability

The RNA-seq data generated in this study are available at Sequence Read Archive (SRA) under the BioProject PRJNA540186.

## Abbreviations

ACC: Acetyl-CoA carboxylase
ADIPOR2: Adiponectin receptor 2
AKT2: AKT serine/threonine kinase 2
AMPK: AMP-activated protein kinase
BF: Backfat thickness
C/EBPA: CCAAT enhancer binding protein alpha
Cas9: CRISPR-associated protein 9
CBE3: Cytosine base editor 3
CRISPR: Clustered regularly interspaced short palindromic repeats
DEGs: Differentially expressed genes
DES: Desmin
DSB: DNA double-strand break
ED: Edited heterozygote—*IGF2*^*C/T*^ pigs
EGFP: Enhanced green fluorescent protein
FABP4: Fatty acid binding protein 4
FACS: Fluorescence-activated cell sorting
FASN: Fatty acid synthase
FPKM: Fragments per kilobase per million
GAS: Gastrocnemius
gRNA: Guide RNA
HDR: Homology-directed repair
HMG-CoA: Hydroxymethylglutaryl-CoA
IGF1: Insulin-like growth factor 1
IGF2: Insulin-like growth factor 2
IGF2R: Insulin like growth factor 2 receptor
IMF: Intramuscular fat
Indels: Insertions and deletions
LGSS: Liang Guang Small Spotted pigs
LL: *Longissimus lumborum*
MSCs: Mesenchymal stem cells
MyHC: Myosin heavy chain
MyoD: Myogenic differentiation
MyoG: Myogenin
NHEJ: Non-homologous end-joining
PAM: Protospacer adjacent motif
PEFs: Porcine embryonic fibroblasts
PI3K-AKT: Phosphatidylinositol 3-kinase-AKT
PM: *Psoas major*
PPARA: Peroxisome proliferator-activated receptor alpha
PPARG: Peroxisome proliferator-activated receptor gamma
qPCR: Quantitative PCR
SCNT: Somatic cell nuclear transfer
SNP: Single nucleotide polymorphism
SREBP1: Sterol regulatory element-binding protein 1
ST: Semitendinosus
WGCNA: Weighted gene co-expression network analysis
WT: Wild type—*IGF2*^*C/C*^ pigs
ZBED6: Zinc finger BED-type containing 6

## References

[1] Oczkowicz M, Tyra M, Ropka-Molik K, Mucha A, Żukowski K (2012). Effect of IGF2 intron3-g.3072G>A on intramuscular fat (IMF) content in pigs raised in Poland. Livest Sci.

[2] Wang X, Xu R, Tong X, Zeng J, Chen M, Lin Z (2022). Characterization of different meat flavor compounds in Guangdong small-ear spotted and Yorkshire pork using two-dimensional gas chromatography-time-of-flight mass spectrometry and multi-omics. LWT.

[3] Giannoukakis N, Deal C, Paquette J, Goodyer CG, Polychronakos C (1993). Parental genomic imprinting of the human IGF2 gene. Nat Genet.

[4] Saltiel AR, Kahn CR (2001). Insulin signalling and the regulation of glucose and lipid metabolism. Nature.

[5] Zanou N, Gailly P (2013). Skeletal muscle hypertrophy and regeneration: interplay between the myogenic regulatory factors (MRFs) and insulin-like growth factors (IGFs) pathways. Cell Mol Life Sci.

[6] Markljung E, Jiang L, Jaffe JD, Mikkelsen TS, Wallerman O, Larhammar M (2009). ZBED6, a novel transcription factor derived from a domesticated DNA transposon regulates IGF2 expression and muscle growth. PLoS Biol.

[7] Andersson L, Andersson G, Hjalm G, Jiang L, Lindblad-Toh K, Lindroth AM (2010). ZBED6: The birth of a new transcription factor in the common ancestor of placental mammals. Transcription.

[8] Van Laere AS, Nguyen M, Braunschweig M, Nezer C, Collette C, Moreau L (2003). A regulatory mutation in IGF2 causes a major QTL effect on muscle growth in the pig. Nature.

[9] Jeon JT, Carlborg O, Törnsten A, Giuffra E, Amarger V, Chardon P (1999). A paternally expressed QTL affecting skeletal and cardiac muscle mass in pigs maps to the IGF2 locus. Nat Genet.

[10] Younis S, Schonke M, Massart J, Hjortebjerg R, Sundstrom E, Gustafson U (2018). The ZBED6-IGF2 axis has a major effect on growth of skeletal muscle and internal organs in placental mammals. Proc Natl Acad Sci U S A.

[11] Younis S, Naboulsi R, Wang X, Cao X, Larsson M, Sargsyan E (2020). The importance of the ZBED6-IGF2 axis for metabolic regulation in mouse myoblast cells. FASEB J.

[12] Xiang G, Ren J, Hai T, Fu R, Yu D, Wang J (2018). Editing porcine IGF2 regulatory element improved meat production in Chinese Bama pigs. Cell Mol Life Sci.

[13] Wang D, Pan D, Xie B, Wang S, Xing X, Liu X (2021). Porcine ZBED6 regulates growth of skeletal muscle and internal organs via multiple targets. PLoS Genet.

[14] Marx V (2018). Base editing a CRISPR way. Nat Methods.

[15] Liu X, Liu H, Wang M, Li R, Zeng J, Mo D (2019). Disruption of the ZBED6 binding site in intron 3 of IGF2 by CRISPR/Cas9 leads to enhanced muscle development in Liang Guang Small Spotted pigs. Transgenic Rec.

[16] Lee JY, Qu-Petersen Z, Cao B, Kimura S, Jankowski R, Cummins J (2000). Clonal isolation of muscle-derived cells capable of enhancing muscle regeneration and bone healing. J Cell Biol.

[17] Pertea M, Pertea GM, Antonescu CM, Chang TC, Mendell JT, Salzberg SL (2015). StringTie enables improved reconstruction of a transcriptome from RNA-seq reads. Nat Biotechnol.

[18] Pertea M, Kim D, Pertea GM, Leek JT, Salzberg SL (2016). Transcript-level expression analysis of RNA-seq experiments with HISAT. StringTie and Ballgown Nat protoc.

[19] Li B, Dewey CN (2011). RSEM: accurate transcript quantification from RNA-Seq data with or without a reference genome. BMC Bioinformatics.

[20] Love MI, Huber W, Anders S (2014). Moderated estimation of fold change and dispersion for RNA-seq data with DESeq2. Genome Biol.

[21] Robinson MD, McCarthy DJ, Smyth GK (2010). edgeR: a Bioconductor package for differential expression analysis of digital gene expression data. Bioinformatics.

[22] Picard B, Lefaucheur L, Berri C, Duclos MJ (2002). Muscle fibre ontogenesis in farm animal species. Reprod Nutr Dev.

[23] Ryu YC, Kim BC (2005). The relationship between muscle fiber characteristics, postmortem metabolic rate, and meat quality of pig longissimus dorsi muscle. Meat Sci.

[24] Glass DJ (2010). PI3 kinase regulation of skeletal muscle hypertrophy and atrophy. Curr Top Microbiol Immunol.

[25] Jiang P, Ren L, Zhi L, Yu Z, Lv F, Xu F (2021). Negative regulation of AMPK signaling by high glucose via E3 ubiquitin ligase MG53. Mol Cell.

[26] Park HS, Min B, Oh SH (2017). Research trends in outdoor pig production - A review. Asian-Australas J Anim Sci.

[27] Brameld JM, Parr T (2016). Improving efficiency in meat production. Proc Nutr Soc.

[28] Zhao JG, Lai LX, Ji WZ, Zhou Q (2019). Genome editing in large animals: current status and future prospects. Natl Sci Rev.

[29] Paquet D, Kwart D, Chen A, Sproul A, Jacob S, Teo S (2016). Efficient introduction of specific homozygous and heterozygous mutations using CRISPR/Cas9. Nature.

[30] Tanihara F, Hirata M, Otoi T (2021). Current status of the application of gene editing in pigs. J Reprod Dev.

[31] Komor AC, Kim YB, Packer MS, Zuris JA, Liu DR (2016). Programmable editing of a target base in genomic DNA without double-stranded DNA cleavage. Nature.

[32] Hernandez-Sanchez J, Amills M, Pena RN, Mercade A, Manunza A, Quintanilla R (2013). Genomic architecture of heritability and genetic correlations for intramuscular and back fat contents in Duroc pigs. J Anim Sci.

[33] Halmos T, Suba I (2019). The physiological role of growth hormone and insulin-like growth factors. Orv Hetil.

[34] Rhoads RP, Baumgard LH, El-Kadi SW, Zhao LD (2016). Physiology and Endocrinology Symposium: Roles for insulin-supported skeletal muscle growth. J Anim Sci.

[35] Du M, Huang Y, Das AK, Yang Q, Duarte MS, Dodson MV (2013). Meat Science and Muscle Biology Symposium: Manipulating mesenchymal progenitor cell differentiation to optimize performance and carcass value of beef cattle. J Anim Sci.

[36] Kruk ZA, Bottema MJ, Reyes-Veliz L, Forder REA, Pitchford WS, Bottema CDK (2018). Vitamin A and marbling attributes: Intramuscular fat hyperplasia effects in cattle. Meat Sci.

[37] Inoki K, Zhu TQ, Guan KL (2003). TSC2 mediates cellular energy response to control cell growth and survival. Cell.

[38] Ahmad B, Serpell CJ, Fong IL, Wong EH (2020). Molecular Mechanisms of Adipogenesis: The Anti-adipogenic Role of AMP-Activated Protein Kinase. Front Mol Biosci.

[39] Lee G, Kim YY, Jang H, Han JS, Nahmgoong H, Park YJ (2022). SREBP1c-PARP1 axis tunes anti-senescence activity of adipocytes and ameliorates metabolic imbalance in obesity. Cell Metab.

[40] Li Y, Xu S, Mihaylova MM, Zheng B, Hou X, Jiang B, et al. AMPK phosphorylates and inhibits SREBP activity to attenuate hepatic steatosis and atherosclerosis in diet-induced insulin-resistant mice. Cell Metab. 2011;13(4):376–88. 10.1016/j.cmet.2011.03.009 PMC3086578 21459323

[41] Smith TM, Cong Z, Gilliland KL, Clawson GA, Thiboutot DM (2006). Insulin-Like Growth Factor-1 Induces Lipid Production in Human SEB-1 Sebocytes Via Sterol Response Element-Binding Protein-1. J Invest Dermatol.

[42] Laubach JP, Fu P, Jiang XH, Salter KH, Potti A, Arcasoy MO (2009). Polycythemia vera erythroid precursors exhibit increased proliferation and apoptosis resistance associated with abnormal RAS and PI3K pathway activation. Exp Hematol.

