# Supplementary material for: Single-base editing in IGF2 improves meat production and intramuscular fat deposition in Liang Guang Small Spotted pigs
Source: J Anim Sci Biotechnol. 2023 Nov 2;14:141. doi: 10.1186/s40104-023-00930-4 (PMC10621156; doi:10.1186/s40104-023-00930-4)
Supplement: Supplementary file 1 — Additional file 1: Table S1. List of primers used in this study. [file 40104_2023_930_MOESM1_ESM.doc]

**Additional file 1: Table S1**

Table S1 List of primers used in this study

| **Primer name** | **Forward (5’→3’)** | **Reverse (5’→3’)** |
| --- | --- | --- |
| **RT-qPCR** | | |
| *GAPDH-*P | ACTCACTCTTCTACCTTTGATGCT | TGTTGCTGTAGCCAAATTCA |
| *IGF2-*P | GTGGCATCGTGGAAGAGTGC | CCAGGTGTCATAGCGGAAGAA |
| *ZBED6-*P | GTACTAGAGCAAAGACTTCCATT | ACAAATTGCCCGCCAGGTAT |
| *MyoD1*-P | TGCCCAAGGTGGAAATCCTG | GCTGTAATAGGTGCCGTCGT |
| *MyoG*-P | GAAAACTACCTGCCCGTCCA | CCACAGACACGGACTTCCTC |
| *MyHC*-P | GCCGACGCTGACAGCGGAAA | AGATGCGGATGCCCTCCA |
| *Cyclin D1*-P | CCGAGAGGCGTCAGAGTTAC | GGGGAGTCTGTCTCCAAACG |
| *Cyclin E1*-P | CAGATGCTGCTAAGGAGGGT | TGATATATGGAACGGGCAGGC |
| *FABP4*-P | GTGGGATGGAAAGTCGACCA | ATCCAGGCCTCTTCCTTTGG |
| *CEBPA*-P | TGGACAAGAACAGCAACGAG | ACCTTCTGTTGAGTCTCCACG |
| *PPARG*-P | CAGCCTCCAGCCCCTCGTC | GCGGTCTCGGCATCTTCTAGG |
| *SREBP1*-P | CGGACGGCTCACAATGC | GCAAGACGGCGGATTTATTC |
| *FASN*-P | TGGATCACTGCATAGACGGC | AGATCCTTGTACACGTCGCC |
| *ACC*-P | TGGAGTTGAACCAGCACTCC | GTAAGGCCAAGCCATCCTGT |
| *HMGcoA*-P | CATGTCAGTTCTCGCCACCT | GCCAGAAGGAGAGCCAAAGT |
| *β-actin*-M | ACGGCCAGGTCATCACTATTG | TGGATGCCACAGGATTCCA |
| *PPARG*-M | GGGGATGTCTCACAATGCCA | GATGGCCACCTCTTTGCTCT |
| *SREBP1*-M | CTCACCATCCTACAGCCTGC | TGCCTTGATGAAGTGTGGCT |
| *FASN*-M | CAGCCCATGCTACAACTGGA | GCACAGTTGCACACCAGAAG |
| *ACC*-M | GGAGGAGGAGGGAAAGGGAT | CTCCCCAAGGAGATACCCCA |
| *HMGcoA*-M | CACGCTCATAGTCGCTGGAT | CAGAAGCCCCAAGCACAAAC |
| **PCR-for genotyping** | | |
| *IGF2*-P | CTTTAAGGAACCAGGTTTTCGCAGC | GTGCTTTGAGGTCTCTGGAAGTTAG |
| **PCR-for detection** | | |
| gRNA-P | GATACAAGGCTGTTAGAGAG | ACGCGCTCCCGCGCTGCGAG |
| EGFP-P | ACAACAAGCACCGGGATAAG | TGCTCAGGTAGTGGTTGTCG |
| **ChIP-qPCR** | | |
| *IGF2*-P | GTGGCATCGTGGAAGAGTGC | CCAGGTGTCATAGCGGAAGAA |

P: pig; M: mouse
