# Supplementary material for: Single-base editing in IGF2 improves meat production and intramuscular fat deposition in Liang Guang Small Spotted pigs
Source: J Anim Sci Biotechnol. 2023 Nov 2;14:141. doi: 10.1186/s40104-023-00930-4 (PMC10621156; doi:10.1186/s40104-023-00930-4)
Supplement: Supplementary file 2 — Additional file 2: Table S2. Antibodies and application used in this study. [file 40104_2023_930_MOESM2_ESM.docx]

**Table S2** Antibodies and application used in this study

| **Antibody name** | **Catalogue Number** | **Brand**  **name** | **Dilution ratio** | | | |
| --- | --- | --- | --- | --- | --- | --- |
|  |  |  | **WB** | **IF** | **ChIP** | **IHC** |
| Anti-GAPDH | G8795 | Sigma | 1:2,000 |  |  |  |
| Anti-IGF2 | ab9574 | Abcam | 1:1,000 |  |  |  |
| Anti-ZBED6 | HPA068807 | ATLAS | 1:1,000 |  | 1:50 |  |
| Anti-IgG | #2729S | Cell Signaling |  |  | 1:50 |  |
| Anti-AKT-S473 | #4058 | Cell Signaling | 1:1,000 |  |  |  |
| Anti-AKT | #9272 | Cell Signaling | 1:1,000 |  |  |  |
| Anti-AMPKα | #2532 | Cell Signaling | 1:1,000 |  |  |  |
| Anti- AMPKα-T172 | #2535 | Cell Signaling | 1:1,000 |  |  |  |
| Anti-SREBP1 | sc-13551 | Santa Cruz | 1:500 |  |  |  |
| Anti-rabbit IgG, HRP-linked Antibody | #7074S | Cell Signaling | 1:500 |  |  |  |
| Anti-mouse IgG, HRP-linked Antibody | #7076S | Cell Signaling | 1:500 |  |  |  |
| Anti-Ki67 | ab15580 | Abcam |  | 1:500 |  |  |
| Anti- Perilipin-1 | ab3526 | Abcam |  | 1:500 |  |  |
| Anti-rabbit IgG(H+L), F(ab')2 Fragment (Alexa Fluor® 488 Conjugate) | #4412S | Cell Signaling |  | 1:500 |  |  |
| Anti-rabbit IgG(H+L), F(ab')2 Fragment (Alexa Fluor® 488 Conjugate) | #4413S | Cell Signaling |  | 1:500 |  |  |
| Anti-Fast Myosin Skeletal Heavy chain | ab51263 | Abcam |  | 1:500 |  | 1:400 |
| Anti-Slow Myosin Skeletal Heavy chain | ab234431 | Abcam |  |  |  | 1:100 |
