# Supplementary material for: Single-base editing in IGF2 improves meat production and intramuscular fat deposition in Liang Guang Small Spotted pigs
Source: J Anim Sci Biotechnol. 2023 Nov 2;14:141. doi: 10.1186/s40104-023-00930-4 (PMC10621156; doi:10.1186/s40104-023-00930-4)
Supplement: Supplementary file 3 — Additional file 3: Fig. S1. Evaluation of gRNA activity and characterization of mutations in single cell clone and edited pigs. [file 40104_2023_930_MOESM3_ESM.docx]

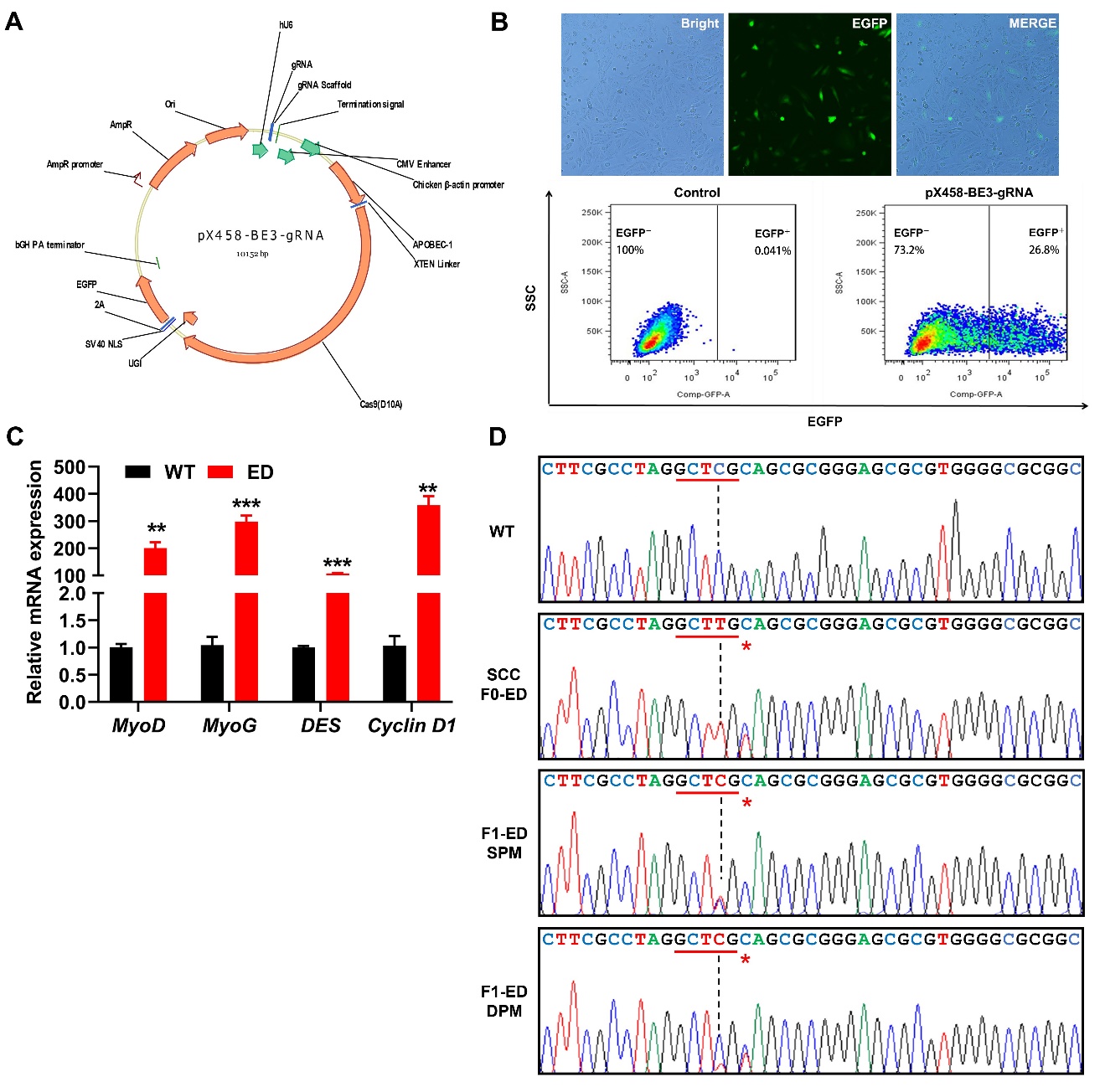


**Fig. S1** Evaluation of gRNA activity and characterization of mutations in single cell clone and edited pigs. **A** The map of pX458-BE3-gRNA expression vector. **B** Image of porcine cells transfected with pX458-BE3-gRNA expressing EGFP, and EGFP positive cells were sorted for subsequent experiments through fluorescence-activated cell sorting (FACS). **C** qPCR analyzed the mRNA levels of critical factors controlling myogenic differentiation in sorted cells from (**B**) and the negative control cells. **D** Sanger sequencing confirmed the existence of desired mutations in selected single cell clone (SCC), founder pigs (F0) and F1 generation pigs. The IGF2-ZBED6 binding motif was labeled with red line. The *IGF2*-intron3-C3071T mutation was indicated by black dashed line, the additional *IGF2*-intron3-C3073T mutation was shown in red asterisk. SPM, Single point mutation; DPM, Double point mutation
