# Supplementary material for: Single-base editing in IGF2 improves meat production and intramuscular fat deposition in Liang Guang Small Spotted pigs
Source: J Anim Sci Biotechnol. 2023 Nov 2;14:141. doi: 10.1186/s40104-023-00930-4 (PMC10621156; doi:10.1186/s40104-023-00930-4)
Supplement: Supplementary file 4 — Additional file 4: Table S3. Summary of generation of IGF2T/T pigs through SCNT. [file 40104_2023_930_MOESM4_ESM.docx]

Table S3 Summary of generation of *IGF2^T/T^* pigs through SCNT

| **No. Experiment** | **Transferred embryos** | **No. Recipients** | **No.**  **Pregnancies, %** | **Litter**  **size** | **No.**  **Born alive** | **No.**  **Healthy piglets** |
| --- | --- | --- | --- | --- | --- | --- |
| 1 | 700 | 5 | 2 (40 %) | 16 | 11 | 8 |
| 2 | 948 | 5 | 1 (20 %) | 6 | 4 | 3 |
| Total | 1,648 | 10 | 3 (30 %) | 22 | 15 | 11 |

A total of 1,648 reconstructed embryos were transferred into 10 surrogates, three surrogates were pregnant and all pregnancies were maintained to term. A total of 22 piglets farrowed, 15 piglets were born alive, 11 healthy piglets survived after 24 h
