# Supplementary material for: Single-base editing in IGF2 improves meat production and intramuscular fat deposition in Liang Guang Small Spotted pigs
Source: J Anim Sci Biotechnol. 2023 Nov 2;14:141. doi: 10.1186/s40104-023-00930-4 (PMC10621156; doi:10.1186/s40104-023-00930-4)
Supplement: Supplementary file 5 — Additional file 5: Table S4. Primer details for detection of off-target sites. [file 40104_2023_930_MOESM5_ESM.docx]

Table S4 Primer details for detection of off-target sites

| **Number** | **Primer sequence (5’→3’)** | **Product length, bp** |
| --- | --- | --- |
| OT1-F | CTTCTCCTGCCACTGAGAGCG | 382 |
| OT1-R | CTCAATTCCCCAAGCAAAACTGG |  |
| OT2-F | CCGGTCCGGCTTAGGAATC | 539 |
| OT2-R | GGTCTTTGACGAAGAGCCGA |  |
| OT3-F | GAACCTCTGCCCTCGTTCC | 570 |
| OT3-R | GACAGCGCGAAAGTTCAGAC |  |

OT, off-target; F, forward; R, reverse
