# Supplementary material for: Single-base editing in IGF2 improves meat production and intramuscular fat deposition in Liang Guang Small Spotted pigs
Source: J Anim Sci Biotechnol. 2023 Nov 2;14:141. doi: 10.1186/s40104-023-00930-4 (PMC10621156; doi:10.1186/s40104-023-00930-4)
Supplement: Supplementary file 6 — Additional file 6: Fig. S2. Off-targeting analysis by Sanger sequencing. [file 40104_2023_930_MOESM6_ESM.docx]

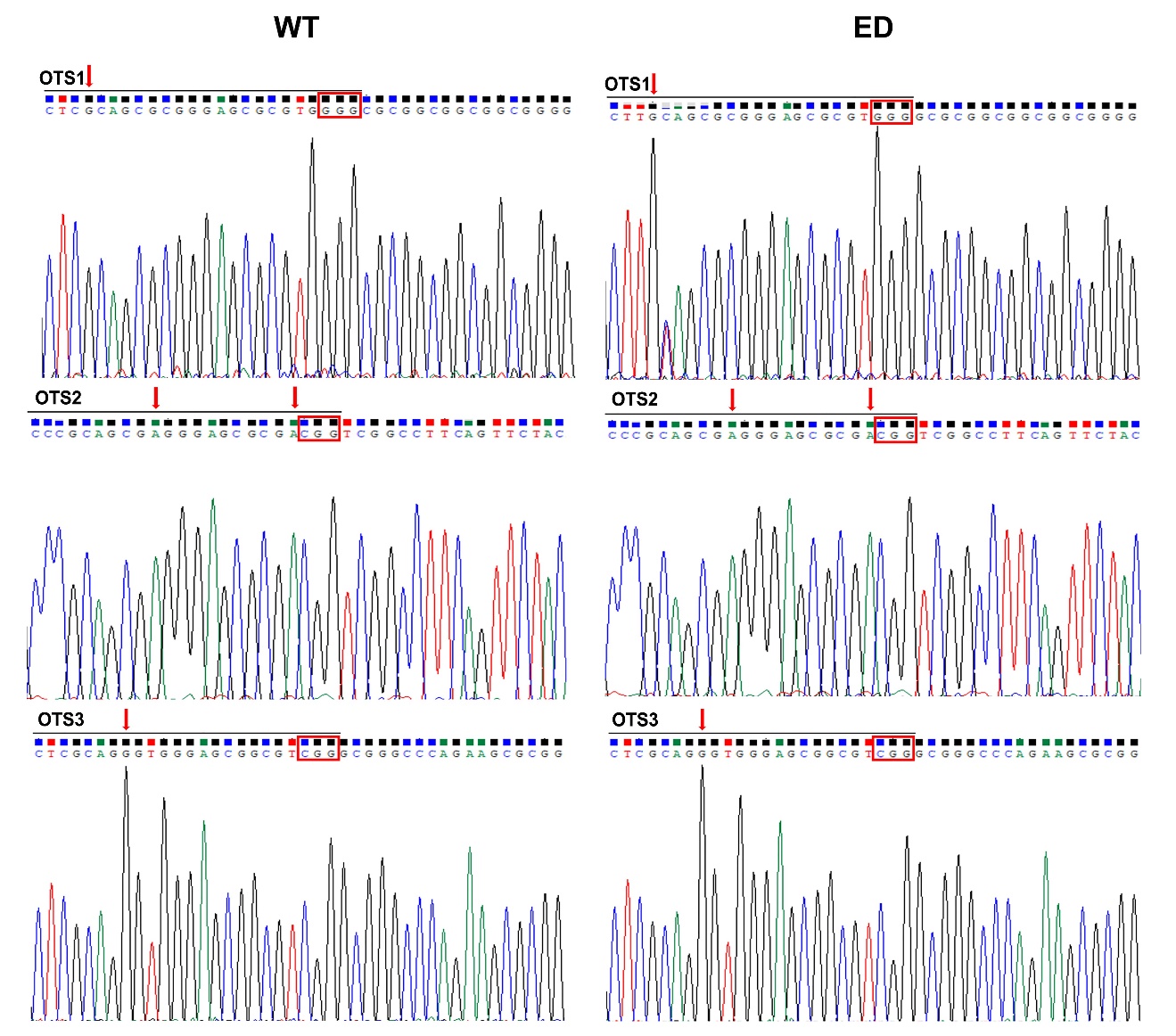


**Fig. S2** Off-targeting analysis by Sanger sequencing. The gray lines represent the predicted off-target sequence, and the red arrows indicate the mismatched sites of sgRNA. The PAM was boxed in red
