# Supplementary material for: Single-base editing in IGF2 improves meat production and intramuscular fat deposition in Liang Guang Small Spotted pigs
Source: J Anim Sci Biotechnol. 2023 Nov 2;14:141. doi: 10.1186/s40104-023-00930-4 (PMC10621156; doi:10.1186/s40104-023-00930-4)
Supplement: Supplementary file 7 — Additional file 7: Fig. S3. Detection of integration of pX458-BE3-gRNA vector in genome of gene-edited pigs. [file 40104_2023_930_MOESM7_ESM.docx]

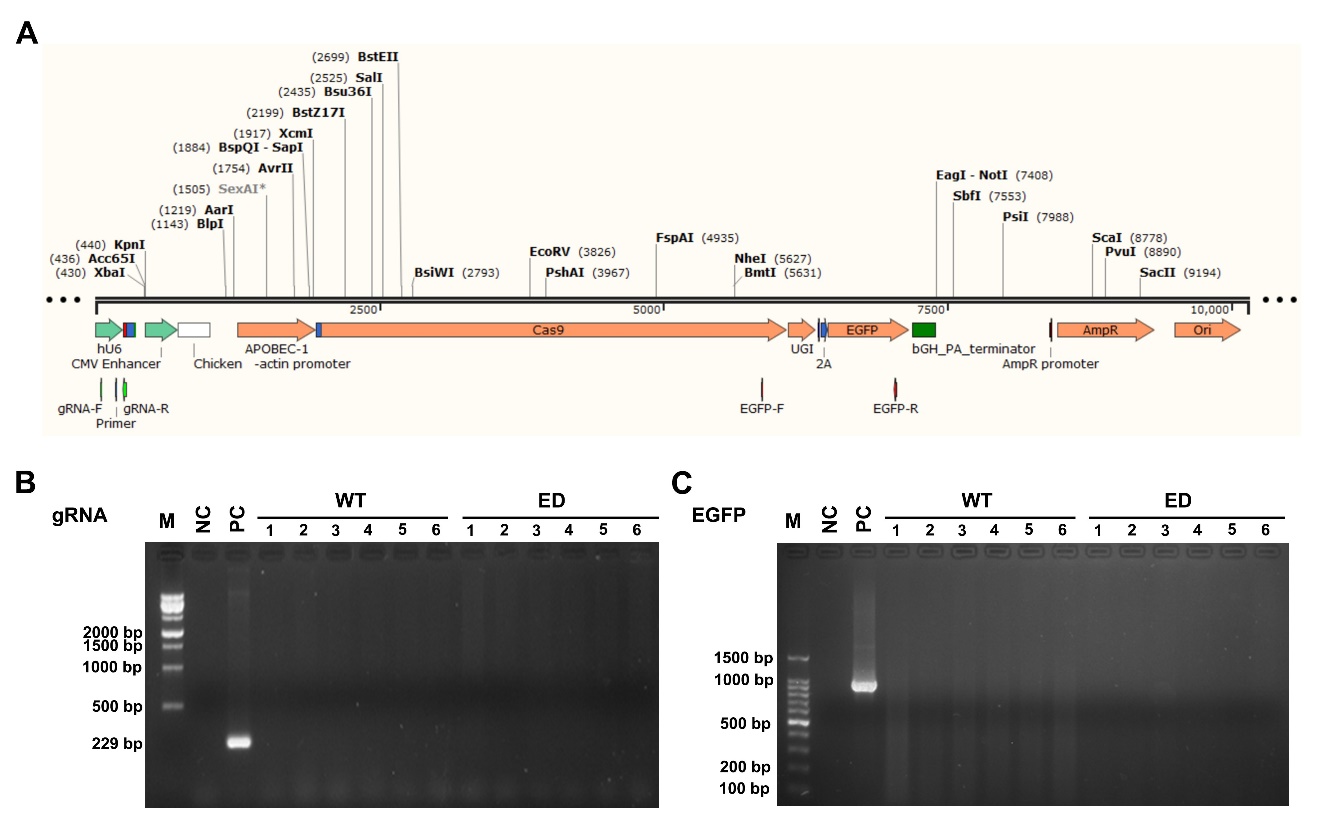


**Fig. S3** Detection of integration of pX458-BE3-gRNA vector in genome of gene-edited pigs. **A** The schematic diagram of pX458-BE3-gRNA vector. The primers used for amplification of gRNA expression cassette or EGFP reporter were indicated. Integration of pX458-BE3-gRNA vector in porcine genome was determined by PCR amplification of gRNA expression cassette **(B)** and **(C)** EGFP reporter on the plasmid from genomic DNA of wild-type and edited pigs. Primers used for PCR detection were listed in Table S1
