# Supplementary material for: Single-base editing in IGF2 improves meat production and intramuscular fat deposition in Liang Guang Small Spotted pigs
Source: J Anim Sci Biotechnol. 2023 Nov 2;14:141. doi: 10.1186/s40104-023-00930-4 (PMC10621156; doi:10.1186/s40104-023-00930-4)
Supplement: Supplementary file 8 — Additional file 8: Fig. S4. Growth phenotype and health detection of IGF2C/T F1 generation pigs. [file 40104_2023_930_MOESM8_ESM.docx]

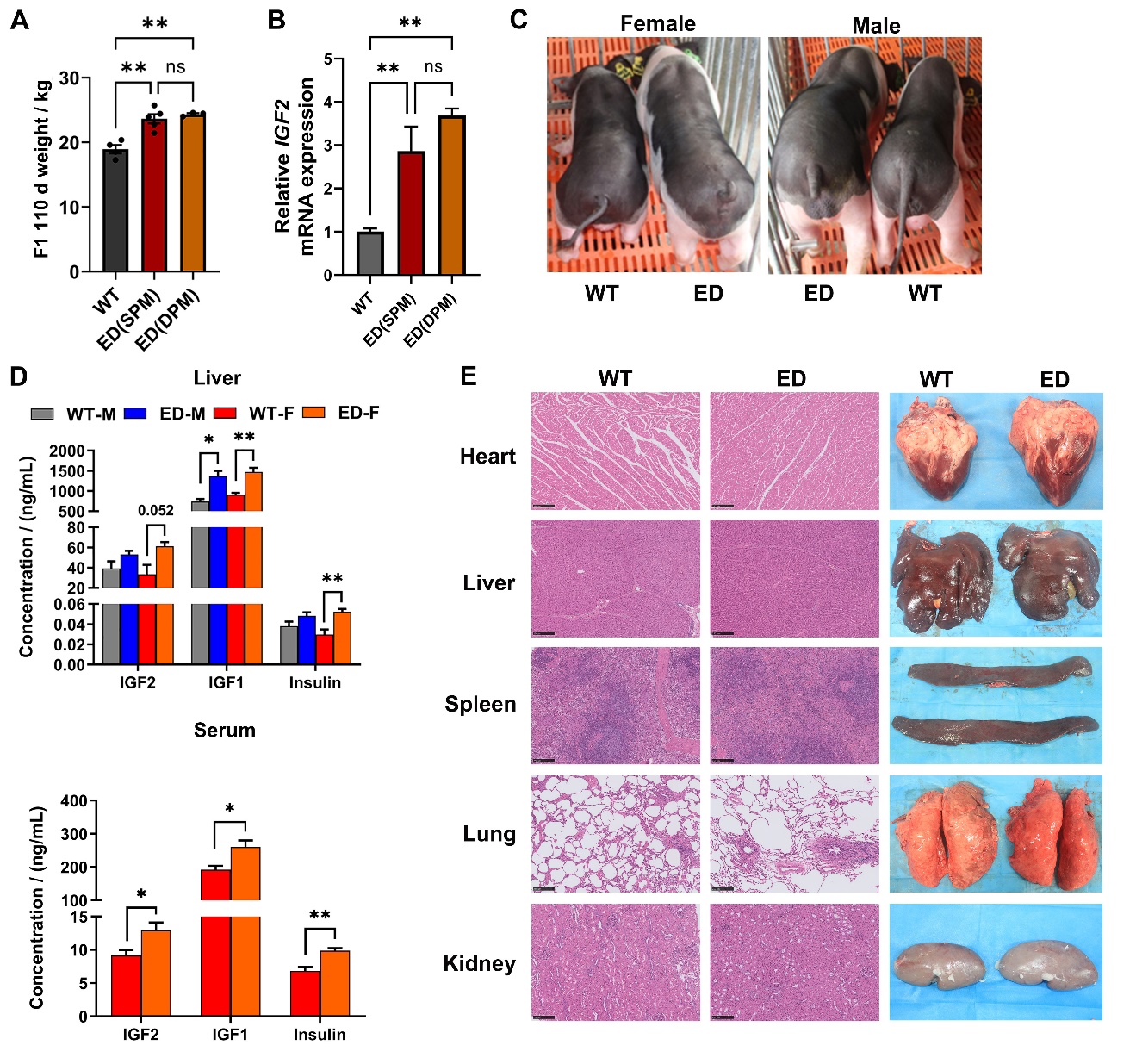


**Fig. S4** Growth phenotype and health detection of *IGF2^C/T^* F1 generation pigs. **A** The body weight and (**B**) the mRNA expression level of *IGF2* of WT, single point mutation (SPM) and double point mutation (DPM) pigs at 110-day-old, with black points representing actual data. **C** Representative photos of WT and *IGF2^C/T^* pigs at age of 3 months, indicating more developed rump of *IGF2^C/T^* pigs. **D** The levels of IGF2, IGF1 and insulin in liver and serum of 370-day-old WT and *IGF2^C/T^* pigs were determined by ELISA (Liver: *n* = 3 per group; Serum: *n* = 4 per group). (**E**) H&E staining of internal organs including heart, liver, spleen, lung, and kidney of 370-day-old WT and *IGF2^C/T^* pigs (Scale bar = 250 μm, *n* = 3 per group). All data were presented as means ± SEM. **P* < 0.05, ***P* < 0.01, ns *P* > 0.05, student’s *t* test
