# Supplementary material for: Single-base editing in IGF2 improves meat production and intramuscular fat deposition in Liang Guang Small Spotted pigs
Source: J Anim Sci Biotechnol. 2023 Nov 2;14:141. doi: 10.1186/s40104-023-00930-4 (PMC10621156; doi:10.1186/s40104-023-00930-4)
Supplement: Supplementary file 9 — Additional file 9: Table S5. Carcass traits and meat quality between WT and IGF2C/T pigs at 270-day-old. [file 40104_2023_930_MOESM9_ESM.docx]

Table S5 Carcass traits and meat quality between WT and *IGF2^C/T^* pigs at 270-day-old

| **Trait**  **270-day-old** | **Male** | | | **Female** | | |
| --- | --- | --- | --- | --- | --- | --- |
|  | **WT pigs *n* = 3** | ***IGF2^C/T^* pigs *n* = 5** | **P-Value** | **WT pigs**  ***n* = 4** | ***IGF2^C/T^* pigs**  ***n* = 4** | ***P*-value** |
| **Carcass traits** | | | | | | |
| Body weight, kg | 43.01±1.62 | 51.86±5.91 | 0.070 | 55.86±3.98 | 64.40±1.34 | 0.020* |
| Carcass weight, kg | 24.58±1.85 | 32.05±2.68 | 0.010* | 37.88±3.70 | 46.08±0.56 | 0.014* |
| Lean mass, kg | 11.14±1.51 | 14.97±1.84 | 0.039* | 12.68±1.06 | 17.24±0.24 | 0.001** |
| Leg and butt, kg | 7.87±0.68 | 10.08±1.46 | 0.077 | 9.80±1.02 | 12.30±0.73 | 0.024* |
| Loin muscle area, cm^2^ | 20.10±2.37 | 20.62±0.77 | 0.664 | 21.34±3.06 | 24.33±2.40 | 0.276 |
| Backfat thickness, mm | 30.46±2.68 | 25.99±4.99 | 0.262 | 29.83±2.49 | 25.08±0.82 | 0.030* |
| Vertical length, cm | 70.67±3.09 | 75.00±1.79 | 0.073 | 70.67±0.47 | 77.50±0.87 | 0.0001*** |
| Slant length, cm | 59.33±2.62 | 65.40±1.36 | 0.010* | 61.67±0.94 | 66.50±1.66 | 0.012* |
| Head weight, kg | 3.40±0.33 | 3.96±0.43 | 0.142 | 4.73±0.09 | 4.45±0.17 | 0.075 |
| Hoof weight, kg | 1.19±0.01 | 1.64±0.15 | 0.004** | 1.40±0.28 | 1.18±0.12 | 0.294 |
| Tail weight, kg | 0.08±0.00 | 0.11±0.02 | 0.025* | 0.09±0.02 | 0.11±0.02 | 0.230 |
| Suet weight, kg | 0.20±0.00 | 0.34±0.07 | 0.034* | 1.93±0.38 | 1.25±0.17 | 0.041* |
| Sebum weight, kg | 6.60±0.43 | 10.70±0.54 | 0.0003*** | 17.67±1.91 | 20.05±0.54 | 0.102 |
| Bone weight, kg | 6.53±0.50 | 8.12±0.32 | 0.003** | 5.47±0.96 | 7.40±0.71 | 0.048* |
| **Meat quality** | | | | | | |
| Meat color, score | 3.67±0.24 | 4.20±0.24 | 0.040* | 2.50±0.41 | 3.33±0.20 | 0.032* |
| Marbling, score | 1.50±0.41 | 2.40±0.37 | 0.033* | 2.50±0.41 | 3.25±0.25 | 0.052 |
| Pressing loss, % | 6.01±0.95 | 6.48±1.32 | 0.656 | 14.51±1.03 | 3.94±2.14 | 0.001** |
| Shear Force, N | 0.90±0.11 | 1.73±0.52 | 0.056 | 0.66±0.14 | 1.05±0.22 | 0.071 |
| pH | 5.95±0.19 | 6.46±0.28 | 0.051 | 5.91±0.14 | 6.54±0.21 | 0.025* |
| **Internal organs** | | | | | | |
| Heart, kg | 0.17±0.01 | 0.20±0.01 | 0.008** | 0.16±0.05 | 0.25±0.01 | 0.031* |
| Liver, kg | 0.66±0.02 | 0.76±0.13 | 0.295 | 0.89±0.02 | 0.86±0.04 | 0.403 |
| Spleen, kg | 0.06±0.01 | 0.07±0.01 | 0.373 | 0.07±0.02 | 0.09±0.01 | 0.109 |
| Lung, kg | 0.44±0.03 | 0.50±0.08 | 0.323 | 0.64±0.18 | 0.50±0.05 | 0.248 |
| Kidney, kg | 0.11±0.01 | 0.13±0.02 | 0.108 | 0.13±0.00 | 0.14±0.01 | 0.376 |

Quantitative data were presented as the mean ± SEM. Significance was established using the student's *t* test. Differences were considered significant at **P* < 0.05, ***P* < 0.01, ****P* < 0.001
