# Supplementary material for: Single-base editing in IGF2 improves meat production and intramuscular fat deposition in Liang Guang Small Spotted pigs
Source: J Anim Sci Biotechnol. 2023 Nov 2;14:141. doi: 10.1186/s40104-023-00930-4 (PMC10621156; doi:10.1186/s40104-023-00930-4)
Supplement: Supplementary file 10 — Additional file 10: Table S6. Carcass traits and meat quality between WT and IGF2C/T pigs at 370-day-old. [file 40104_2023_930_MOESM10_ESM.docx]

Table S6 Carcass traits and meat quality between WT and *IGF2^C/T^* pigs at 370-day-old

| **Trait**  **370-day-old** | **Male** | | | **Female** | | |
| --- | --- | --- | --- | --- | --- | --- |
|  | **WT pigs *n* = 4** | ***IGF2^C/T^* pigs *n* = 4** | ***P*-value** | **WT pigs**  ***n* = 4** | ***IGF2^C/T^* pigs**  ***n* = 4** | ***P*-value** |
| **Carcass traits** | | | | | | |
| Body weight, kg | 95.60±7.28 | 98.01±6.93 | 0.692 | 83.23±1.06 | 91.60±7.58 | 0.107 |
| Carcass weight, kg | 65.38±7.23 | 69.45±5.09 | 0.456 | 56.94±1.18 | 66.99±6.89 | 0.047* |
| Lean mass, kg | 20.50±0.96 | 24.17±1.18 | 0.006** | 18.54±0.61 | 23.60±1.82 | 0.004** |
| Leg and butt, kg | 16.65±1.24 | 17.05±3.07 | 0.841 | 14.35±0.38 | 17.30±1.92 | 0.040* |
| Loin muscle area, cm^2^ | 83.25±6.94 | 88.13±1.24 | 0.276 | 83.50±2.06 | 83.75±5.89 | 0.947 |
| Backfat thickness, mm | 23.79±1.43 | 26.04±0.66 | 0.049* | 22.36±1.01 | 26.19±0.95 | 0.003** |
| Vertical length, cm | 43.50±8.02 | 37.37±5.68 | 0.321 | 38.00±3.67 | 36.21±3.72 | 0.576 |
| Slant length, cm | 70.50±6.58 | 75.75±1.79 | 0.230 | 71.25±0.83 | 74.13±3.78 | 0.246 |
| Head weight, kg | 5.40±0.32 | 6.95±1.31 | 0.093 | 5.40±0.37 | 6.25±0.46 | 0.047* |
| Hoof weight, kg | 1.65±0.17 | 1.70±0.30 | 0.824 | 1.60±0.14 | 1.56±0.08 | 0.646 |
| Tail weight, kg | 0.12±0.02 | 0.12±0.01 | 0.637 | 0.09±0.02 | 0.11±0.01 | 0.138 |
| Suet weight, kg | 3.65±0.43 | 3.23±0.47 | 0.300 | 2.45±0.30 | 2.34±0.43 | 0.728 |
| Sebum weight, kg | 32.80±5.45 | 31.95±3.51 | 0.828 | 28.40±1.03 | 29.55±4.19 | 0.661 |
| Bone weight, kg | 8.10±0.46 | 9.75±1.76 | 0.167 | 7.25±0.22 | 8.00±1.07 | 0.278 |
| **Meat quality** | | | | | | |
| Meat color, score | 2.00±0.71 | 3.25±0.43 | 0.040* | 2.25±0.43 | 3.25±0.43 | 0.030* |
| Marbling, score | 2.00±0.71 | 2.88±0.22 | 0.086 | 1.75±0.71 | 2.75±0.22 | 0.013* |
| Pressing loss, % | 13.39±8.63 | 4.35±2.52 | 0.132 | 15.66±7.91 | 4.94±2.67 | 0.068 |
| Shear Force, N | 0.66±0.14 | 1.05±0.22 | 0.267 | 0.78±0.21 | 1.41±0.16 | 0.006** |
| pH | 6.17±0.31 | 6.77±0.18 | 0.027* | 6.07±0.32 | 6.69±0.30 | 0.050 |
| **Internal organs** | | | | | | |
| Heart, kg | 0.26±0.03 | 0.33±0.01 | 0.025* | 0.23±0.02 | 0.28±0.03 | 0.031* |
| Liver, kg | 1.16±0.17 | 1.13±0.08 | 0.807 | 0.96±0.05 | 1.11±0.25 | 0.318 |
| Spleen, kg | 0.14±0.02 | 0.11±0.01 | 0.128 | 0.11±0.01 | 0.11±0.03 | 0.903 |
| Lung, kg | 0.62±0.09 | 0.62±0.16 | 0.970 | 0.52±0.06 | 0.63±0.04 | 0.050 |
| Kidney, kg | 0.33±0.06 | 0.35±0.14 | 0.892 | 0.30±0.02 | 0.35±0.07 | 0.295 |

Quantitative data were presented as the mean ± SEM. Significance was established using the student's *t* test. Differences were considered significant at **P* < 0.05 ***P* < 0.01, ****P* < 0.001
