# Supplementary material for: Single-base editing in IGF2 improves meat production and intramuscular fat deposition in Liang Guang Small Spotted pigs
Source: J Anim Sci Biotechnol. 2023 Nov 2;14:141. doi: 10.1186/s40104-023-00930-4 (PMC10621156; doi:10.1186/s40104-023-00930-4)
Supplement: Supplementary file 11 — Additional file 11: Table S7. Blood routine test of WT and IGF2C/T pigs at 270-day-old. [file 40104_2023_930_MOESM11_ESM.docx]

Table S7 Blood routine test of WT and IGF2^C/T^ pigs at 270-day-old

| **Blood routine indexes** | **Male** | | | **Female** | | |
| --- | --- | --- | --- | --- | --- | --- |
|  | **WT pigs**  ***n* = 5** | ***IGF2^C/T^* pigs *n* = 9** | ***P*-value** | **WT pigs**  ***n* = 9** | ***IGF2^C/T^* pigs**  ***n* = 10** | ***P*-value** |
| WBC, 10^9^/L | 19.56±0.61 | 16.71±5.76 | 0.327 | 22.07±3.84 | 19.16±2.65 | 0.075 |
| Neu, 10^9^/L | 5.00±0.56 | 4.08±2.20 | 0.410 | 5.86±1.45 | 5.00±1.04 | 0.158 |
| Lym, 10^9^/L | 13.90±0.40 | 11.58±3.40 | 0.185 | 15.59±3.10 | 13.67±2.16 | 0.141 |
| Mon, 10^9^/L | 0.55±0.04 | 0.95±0.68 | 0.252 | 0.47±0.48 | 0.34±0.25 | 0.456 |
| Eos, 10^9^/L | 0.08±0.05 | 0.07±0.03 | 0.885 | 0.09±0.08 | 0.11±0.11 | 0.647 |
| Bas, 10^9^/L | 0.03±0.02 | 0.02±0.02 | 0.748 | 0.05±0.02 | 0.05±0.02 | 0.291 |
| Neu, % | 25.52±2.32 | 23.20±6.13 | 0.466 | 26.56±3.99 | 26.02±3.52 | 0.765 |
| Lym, % | 71.10±1.93 | 70.37±5.59 | 0.797 | 70.49±4.76 | 71.41±4.49 | 0.679 |
| Mon, % | 2.84±0.26 | 5.86±3.01 | 0.061 | 2.30±2.52 | 1.76±1.26 | 0.565 |
| Eos, % | 0.40±0.28 | 0.43±0.11 | 0.775 | 0.42±0.44 | 0.55±0.52 | 0.571 |
| Bas, % | 0.14±0.10 | 0.14±0.07 | 0.930 | 0.23±0.08 | 0.25±0.14 | 0.703 |
| RBC, 10^12^/L | 7.16±0.56 | 7.92±0.27 | 0.008** | 6.80±0.57 | 6.98±0.76 | 0.595 |
| HGB, g/L | 136.80±4.83 | 134.11±5.07 | 0.388 | 126.00±15.03 | 127.64±14.48 | 0.817 |
| HCT, % | 43.88±2.88 | 45.06±1.88 | 0.411 | 39.09±5.54 | 39.45±5.57 | 0.894 |
| MCV, fL | 59.76±3.23 | 56.74±1.35 | 0.043* | 59.33±7.05 | 61.22±2.20 | 0.437 |
| MCH, pg | 18.22±1.06 | 16.74±0.40 | 0.005** | 18.89±1.29 | 18.84±1.01 | 0.924 |
| MCHC, g/L | 303.20±15.25 | 295.67±6.58 | 0.258 | 300.56±6.85 | 303.18±8.00 | 0.470 |
| RDW-CV, % | 19.66±1.59 | 17.74±0.91 | 0.021* | 20.78±1.84 | 21.69±5.09 | 0.633 |
| PLT, 10^9^/L | 288.80±29.18 | 278.89±82.44 | 0.814 | 263.00±83.72 | 292.36±90.22 | 0.487 |
| MPV, fL | 8.16±0.29 | 8.51±0.77 | 0.380 | 9.26±0.94 | 9.19±0.92 | 0.885 |
| PDW, % | 11.94±1.48 | 11.89±1.37 | 0.953 | 14.16±2.03 | 13.55±1.77 | 0.505 |
| PCT, % | 0.24±0.02 | 0.26±0.07 | 0.607 | 0.25±0.07 | 0.29±0.07 | 0.285 |

WBC, white blood cell; Neu, neutrophil; Lym, lymphocyte; Mon, monocyte; Eos, eosinophils; Bas, basophils; Neu%, percentage of neutrophils; Lym%, percentage of lymphocytes; Mon%, percentage of monocytes; Eos%, percentage of eosinophils; Bas%, percentage of basophils; RBC, red blood cell; HGB, hemoglobin; HCT, hematocrit; MCV, mean corpuscular volume; MCH, mean corpuscular hemoglobin; MCHC, mean corpuscular hemoglobin concentration; RDW-CV, red cell distribution width-variable coefficient; PLT, platelet; MPV, mean platelet volume; PDW, platelet distribution width; PCT, plateletcrit. Quantitative data were presented as the mean ± SEM. Significance was established using the student's *t* test. Differences were considered significant at **P* < 0.05 and ***P* < 0.01
