# Supplementary material for: Single-base editing in IGF2 improves meat production and intramuscular fat deposition in Liang Guang Small Spotted pigs
Source: J Anim Sci Biotechnol. 2023 Nov 2;14:141. doi: 10.1186/s40104-023-00930-4 (PMC10621156; doi:10.1186/s40104-023-00930-4)
Supplement: Supplementary file 12 — Additional file 12: Table S8. Blood biochemistry test of WT and IGF2C/T pigs at 270-day-old. [file 40104_2023_930_MOESM12_ESM.docx]

Table S8 Blood biochemistry test of WT and *IGF2^C/T^* pigs at 270-day-old

| **Blood biochemical**  **indexes** | **Male** | | | **Female** | | |
| --- | --- | --- | --- | --- | --- | --- |
|  | **WT pigs**  ***n* = 5** | ***IGF2^C/T^* pigs *n* = 9** | ***P*-value** | **WT pigs**  ***n* = 9** | ***IGF2^C/T^* pigs**  ***n* = 10** | ***P*-value** |
| ALT, U/L | 54.20±8.01 | 53.56±8.80 | 0.902 | 36.56±6.95 | 57.67±5.77 | 5.99E-06*** |
| AST, U/L | 109.00±60.85 | 128.78±91.88 | 0.696 | 33.11±9.07 | 42.11±19.75 | 0.259 |
| TP, g/L | 75.82±2.03 | 81.28±5.56 | 0.073 | 73.82±3.78 | 70.47±5.95 | 0.189 |
| ALB, g/L | 36.04±1.62 | 33.91±1.57 | 0.046* | 31.77±1.95 | 32.70±2.89 | 0.450 |
| GLOB, g/L | 39.78±2.80 | 47.37±5.34 | 0.018* | 42.06±2.85 | 37.77±3.61 | 0.015* |
| A/G | 0.91±0.09 | 0.72±0.09 | 0.005** | 0.76±0.06 | 0.87±0.07 | 0.003** |
| TBIL, μmol/L | 2.25±0.27 | 1.97±0.20 | 0.067 | 2.31±0.15 | 2.18±0.11 | 0.061 |
| DBIL, μmol/L | 0.67±0.11 | 0.59±0.13 | 0.305 | 0.68±0.08 | 0.70±0.07 | 0.607 |
| IBIL, μmol/L | 1.57±0.30 | 1.37±0.16 | 0.164 | 1.63±0.13 | 1.53±0.09 | 0.089 |
| D/T | 0.31±0.06 | 0.30±0.06 | 0.871 | 0.29±0.03 | 0.32±0.03 | 0.136 |
| ALP, U/L | 128.00±25.34 | 126.11±38.61 | 0.929 | 95.89±29.44 | 113.44±20.91 | 0.188 |
| TBA, μmol/L | 6.50±0.39 | 15.77±7.94 | 0.033* | 6.83±0.68 | 6.84±0.51 | 0.982 |
| BUN, mmol/L | 2.65±0.39 | 2.20±0.42 | 0.095 | 2.95±0.48 | 3.16±0.77 | 0.515 |
| CR, μmol/L | 136.72±21.90 | 98.99±38.02 | 0.083 | 143.28±27.96 | 135.24±23.24 | 0.541 |
| UA, μmol/L | 21.20±1.94 | 18.56±2.27 | 0.064 | 26.00±2.31 | 22.56±2.22 | 0.008** |
| TG, mmol/L | 0.65±0.07 | 0.55±0.13 | 0.191 | 0.57±0.08 | 0.55±0.07 | 0.603 |
| TC, mmol/L | 1.23±0.15 | 1.45±0.33 | 0.236 | 2.14±0.35 | 2.05±0.33 | 0.607 |
| HDL-C, mmol/L | 0.54±0.08 | 0.57±0.12 | 0.646 | 0.75±0.15 | 0.81±0.14 | 0.428 |
| LDL-C, mmol/L | 0.41±0.07 | 0.57±0.18 | 0.094 | 1.02±0.26 | 0.89±0.17 | 0.249 |
| GLU, mmol/L | 2.55±0.42 | 3.09±0.65 | 0.149 | 3.47±0.66 | 2.59±0.53 | 0.008** |
| CK, U/L | 2,293.40±1726.12 | 2,265.56±1273.98 | 0.975 | 253.13±134.46 | 279.17±131.71 | 0.743 |
| LDH, U/L | 775.60±250.97 | 964.89±364.24 | 0.357 | 533.56±127.32 | 523.89±138.76 | 0.886 |
| Ca, mmol/L | 2.91±0.07 | 2.69±0.11 | 0.003** | 2.70±0.06 | 2.71±0.11 | 0.776 |

ALT, alanine aminotransferase; AST, aspartate aminotransferase; TP, total protein; ALB, albumin; GLOB, globulin; A/G, albumin/globulin; TBIL, total bilirubin; DBIL, direct bilirubin; IBIL, indirect bilirubin; D/T, DBIL/TBIL; ALP, alkaline phosphatase; TBA, total bile acid; BUN, blood urea nitrogen; CR, creatinine; UA, uric acid; TG, triglyceride; TC, total cholesterol; HDL-C, high density lipoprotein cholesterol; LDL-C, low density lipoprotein cholesterol; GLU, glucose; CK, creatine kinase; LDH, lactic dehydrogenase; Ca, calcium. Quantitative data were presented as the mean ± SEM. Significance was established using the student's *t* test. Differences were considered significant at **P* < 0.05 and ***P* < 0.01
