# Supplementary material for: Single-base editing in IGF2 improves meat production and intramuscular fat deposition in Liang Guang Small Spotted pigs
Source: J Anim Sci Biotechnol. 2023 Nov 2;14:141. doi: 10.1186/s40104-023-00930-4 (PMC10621156; doi:10.1186/s40104-023-00930-4)
Supplement: Supplementary file 13 — Additional file 13: Fig. S5. Identification of modules correlated to intramuscular fat content by weighted gene correlation network analysis (WGCNA). [file 40104_2023_930_MOESM13_ESM.docx]

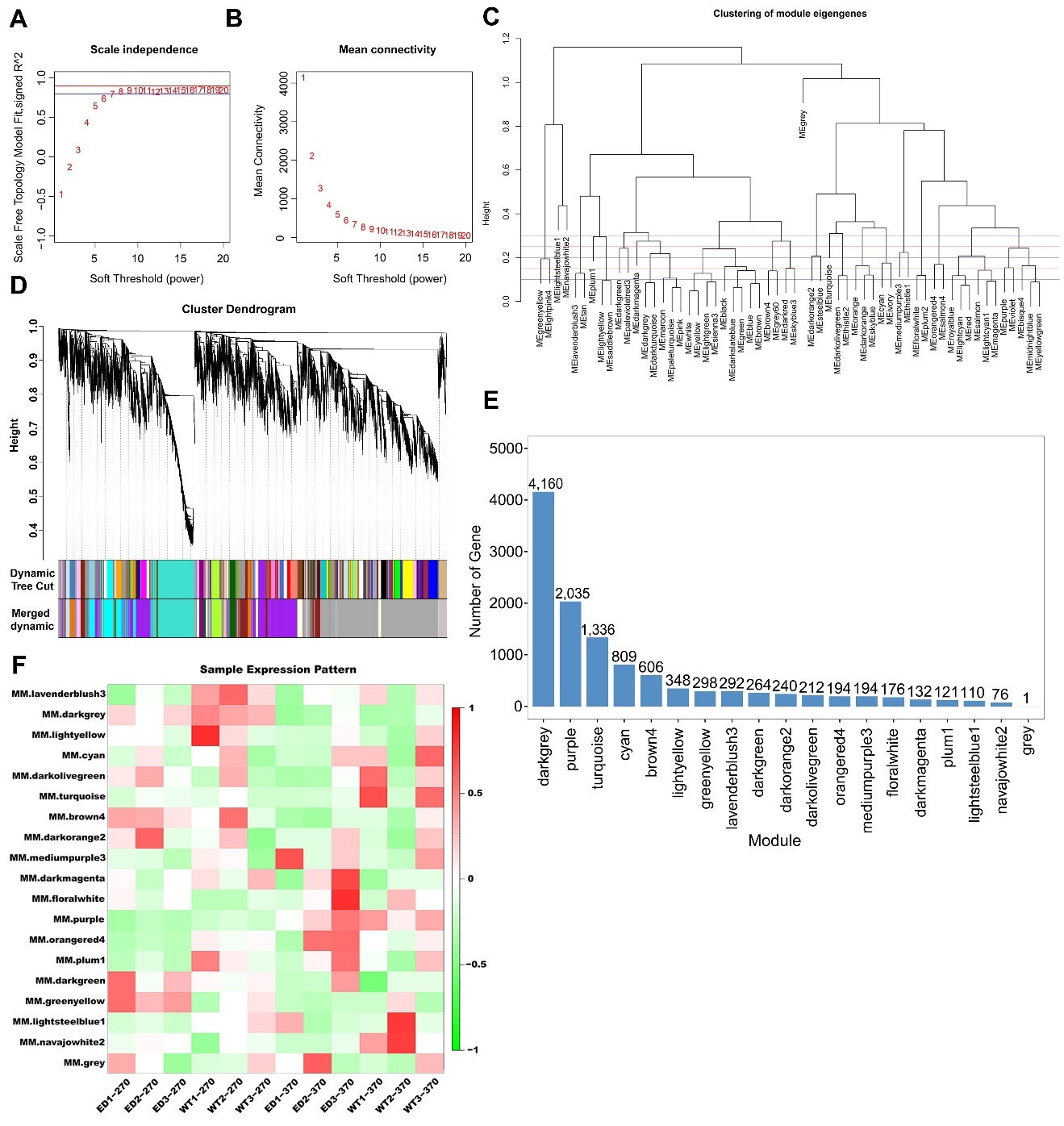


**Fig. S5** Identification of modules correlated to intramuscular fat content by weighted gene correlation network analysis (WGCNA). A network topology analysis for adjacency matrices with different soft threshold powers. Red numbers indicated the soft-threshold power corresponding to the correlation coefficient square value in (**A**) and mean connectivity in (**B**). **C** The gene cluster tree was constructed according to the correlation between gene expression levels. The similarity degree selected was 0.75, and the minimum number of genes selected for analysis was 50, in this analysis. **D** The clustering dendrogram and expression heatmap of genes were identified by WGCNA. **E** The number of genes in each of the 19 identified modules. **F** The correlation of the identified modules with the intramuscular fat content in each sample
