# Supplementary material for: Single-base editing in IGF2 improves meat production and intramuscular fat deposition in Liang Guang Small Spotted pigs
Source: J Anim Sci Biotechnol. 2023 Nov 2;14:141. doi: 10.1186/s40104-023-00930-4 (PMC10621156; doi:10.1186/s40104-023-00930-4)
Supplement: Supplementary file 14 — Additional file 14: Fig. S6. qPCR analysis of the relative transcription levels of downstream target genes in AMPK signaling pathway. [file 40104_2023_930_MOESM14_ESM.docx]

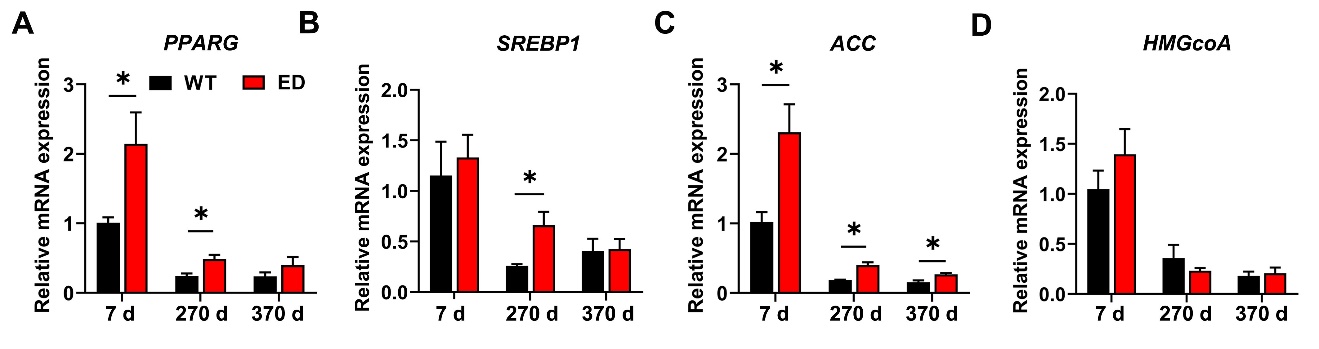


**Fig. S6** qPCR analyzed the relative transcription levels of downstream target genes in AMPK signaling pathway. Samples from the *longissimus lumborum* muscle of 7, 270 and 370-day-old WT and *IGF2^C/T^* pigs. *n* = 3-4 per group. All data were presented as means ± SEM. **P* < 0.05, student’s *t* test
