# Supplementary material for: Single-base editing in IGF2 improves meat production and intramuscular fat deposition in Liang Guang Small Spotted pigs
Source: J Anim Sci Biotechnol. 2023 Nov 2;14:141. doi: 10.1186/s40104-023-00930-4 (PMC10621156; doi:10.1186/s40104-023-00930-4)
Supplement: Supplementary file 15 — Additional file 15: Fig. S7. Overexpression of IGF2 in 3T3-L1 cells promoted cell proliferation and adipogenic differentiation through the PI3K-AKT/AMPK pathway. [file 40104_2023_930_MOESM15_ESM.docx]

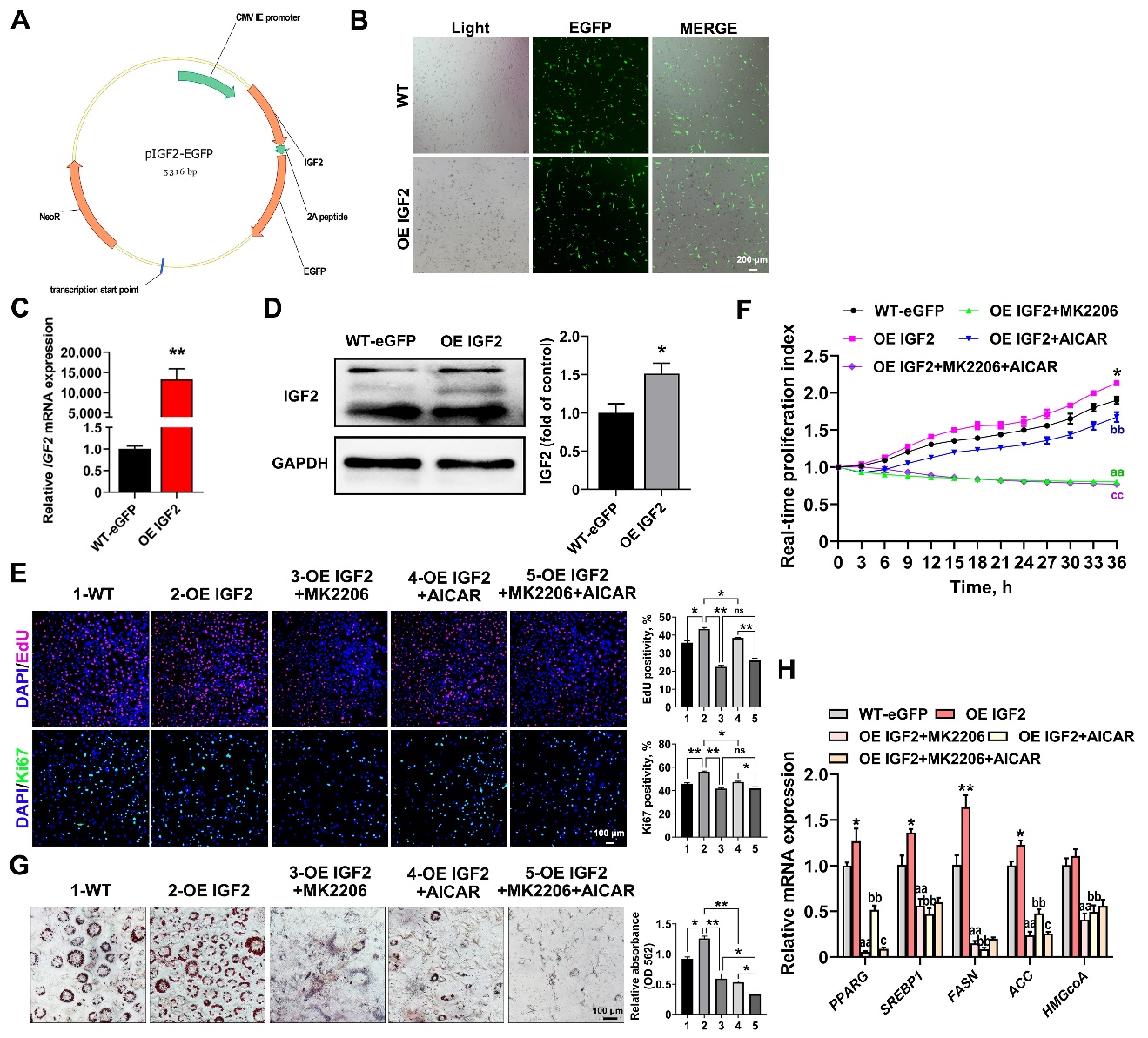


**Fig. S7** Overexpression of *IGF2* in 3T3-L1 cells promoted cell proliferation and adipogenic differentiation through the PI3K-AKT/AMPK pathway. **A** Schematic diagram of *IGF2-2A-EGFP* expression plasmid. **B** Images of 3T3-L1 cells 48 h post transfection of *IGF2-2A-EGFP* expression plasmid. Scale bar = 200 μm. **C** The mRNA expression and (**D**) protein levels of *IGF2* were detected after overexpression in 3T3-L1 cells. **E** EdU and Ki67 immunofluorescent staining were performed between WT-eGFP cell, OE IGF2 cell, and OE IGF2 cell with the treat of MK2206, AICAR, or MK2206 + AICAR. The percentage of EdU-positive or Ki67-positive cells were presented as histogram in the right panel. Scale bar = 100 μm. **F** Real-time cell proliferation monitoring assay was carried out to measure the proliferation index of the above 5 groups, *n* = 3 per group. Mark of significance, *: WT-eGFP vs. OE IGF2; a: OE IGF2 vs. OE IGF2+MK2206; b: OE IGF2 vs. OE IGF2+AICAR; c: OE IGF2+AICAR vs. OE IGF2+MK2206+AICAR. *P* < 0.05 and *P* < 0.01 significance was reached from 15 h to 36 h, only 36 h was marked in the figure. **G** Oil red O staining of lipid droplet formation at day 6 of induced adipogenic differentiation in the above 5 groups. The absorption value of triglyceride content was presented as histogram in the right panel. Scale bar = 100 μm. **H** qPCR analyzed the relative transcription levels of downstream target genes in AMPK signaling pathway. Mark of significance was same as (*F*). All data were presented as means ± SEM. *n* = 3-4 per group. ^*^*P* < 0.05, ^**^*P* < 0.01, ^***^*P* < 0.001, student’s *t* test, one-way ANOVA or two-way ANOVA
